# Synthetic integrin-binding immune stimulators target cancer cells and prevent tumor formation

**DOI:** 10.1038/s41598-017-17627-0

**Published:** 2017-12-14

**Authors:** Manuel Brehs, André J. G. Pötgens, Julia Steitz, Karine Thewes, Janett Schwarz, Anne C. Conibear, Matthias Bartneck, Frank Tacke, Christian F. W. Becker

**Affiliations:** 1 Institute of Biological Chemistry, Faculty of Chemistry, University of Vienna, 1090 Vienna, Austria; 2 Syntab Therapeutics GmbH, 52074 Aachen, Germany; 3 Institute for Laboratory Animal Science, University Hospital RWTH, Aachen, Germany; 4 Department of Medicine III, University Hospital RWTH, Aachen, Germany

## Abstract

Immuno-oncology approaches mainly utilize monoclonal antibodies or protein-based scaffolds that bind with high affinity to cancer cells and can generate an immune response. Peptides can also bind with high affinity to cancer cells and are intermediate in size between antibodies and small molecules. They are also synthetically accessible and therefore easily modified to optimize their stability, binding affinity and selectivity. Here we describe the design of immune system engagers (ISErs), a novel class of synthetic peptide-based compounds that bind specifically to cancer cells and stimulate the immune system. A prototype, Y9, targets integrin α_3_, which is overexpressed on several cancer cells, and activates the immune system via a formyl methionine-containing effector peptide. Injection of Y9 leads to immune cell infiltration into tissue and prevents tumor formation in a guinea pig model. The anti-tumor activity and synthetic accessibility of Y9 illustrate that ISErs could be applied to a wide variety of targets and diseases.

## Introduction

Antibodies (Abs), antibody-drug conjugates and their derivatives have become a significant part of state of the art cancer treatments^1^. More than 14 monoclonal antibodies (mAbs) are currently approved for cancer therapy and many more are under development^2–4^. These antibodies are raised against unique cell surface receptors or antigens, allowing specific targeting of tumor cells.

Immune-mediated mechanisms, including complement-dependent cytotoxicity (CDC), antibody-dependent cell-mediated cytotoxicity (ADCC) and other secondary immunological effects have been shown to play a crucial role in the therapeutic efficacy of mAbs^2,5,6^. The mechanism of action of mAbs was previously thought to involve blocking the physiological function of the target (e.g. a growth factor or cytokine receptor) by the Fab portion but recent studies have demonstrated stimulation of the immune system by the Fc portion; Fc gamma receptor-mediated activation of macrophages and natural killer cells leading to ADCC is necessary for the anti-tumor effects of Rituximab (anti-CD20) and Trastuzumab/Herceptin (anti-Her2/ErbB2)^7^.

Both biological and chemical approaches have been developed to overcome the intrinsic limitations of mAbs with respect to selectivity, large size, stability, limited scope for alterations and production costs. Several approaches based on full IgGs, antibody fragments or antibody mimics have been described^8–19^ and multispecificity has been achieved via genetic fusion or by chemical cross-linking of different complementarity determining regions (CDRs)^20,21^. A small molecule approach utilizes antibody-recruiting molecules (ARMs) that combine a target-binding moiety with one for antibody recruitment^22^. Recently, in a combination of biological and chemical methods, synthetic peptides have been linked to antibody scaffolds using site-selective reactions^11,23^ and a synthetic molecule with targeting and effector functions similar to those of antibodies has also been reported^24^.

**Figure 1:**
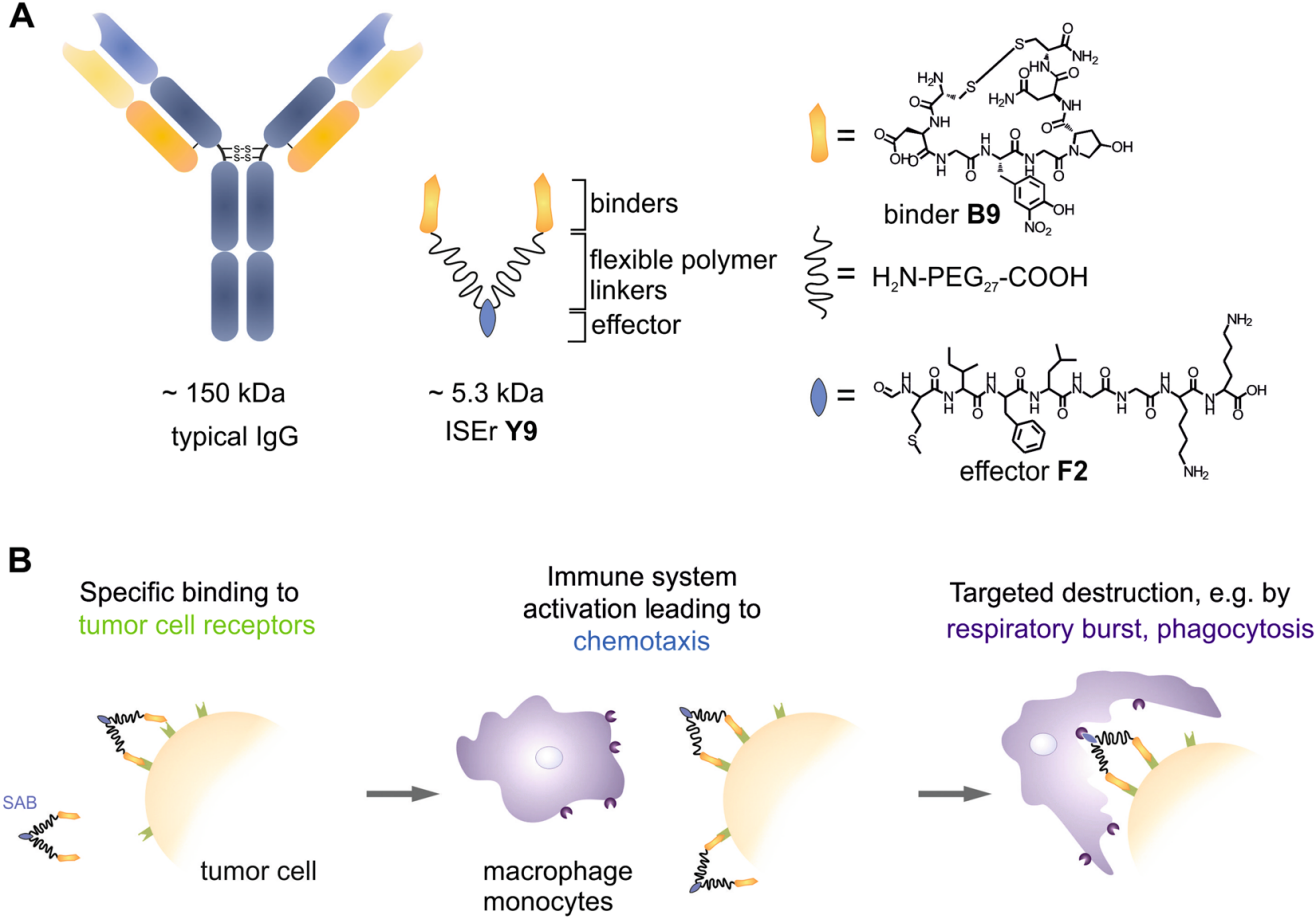
(**A**) Comparison of a typical IgG (left) and the synthetic immune system engager (ISEr) **Y9** consisting of an effector (**F**) as well as two linker and binder (**B**) moieties. (**B**) Proposed mechanism of innate immune system activation by **Y9**. Upon specific binding to tumor cells, neutrophils, monocytes and macrophages are recruited to these cells and directly attack tumor cells displaying **Y9**. Further effects are based on cytokine release of macrophages and monocytes, recruiting other immune cells.

We sought to design a fully synthetic molecule of intermediate size (~5 kDa) between small molecules and Abs that would harness the ability of Abs to recognize tumor cells and initiate an innate immune response against them. These immune system engagers (ISEr) comprise an immune stimulatory effector peptide and two binder peptides that bind selectively to cell-surface markers of tumor or tumor-associated cells, are synthetically accessible and do not activate cell-surface receptors (Fig. 1). The two binder peptides are linked to the effector peptide via chemically inert, non-immunogenic, monodisperse polyethylene glycol (PEG) chains. The PEG length (10 nm per PEG_27_^25^) was chosen to cover similar distances as the two paratopes in an antibody (Fig. 1A). By combining two binder peptides per ISEr, we aimed to avoid the fast dissociation and low retention times that can reduce the efficacy of even high-affinity monovalent binders under non-equilibrium physiological conditions^26^.

We selected an immune stimulatory effector peptide based on an N-formyl methionine containing peptide employed to activate the innate immune system via granulocytes, monocytes and macrophages (denoted as **F1** or **F2**, Fig. 1). Such peptides are typically of bacterial origin and are well-known to elicit an innate immune response by interaction with various immune cell receptors such as three members of the N-formyl-peptide receptor family (FPR1–3)^27–29^. Previous experiments in which a formyl-methionyl-leucyl-phenylalanine peptide (fMLF) was covalently linked to an IgG antibody induced monocyte chemotaxis^30^ and a two-fold increase in macrophage infiltration of hepatomas and a decrease in tumor weight in guinea pigs^31^. At the same time no relevant toxicity in a human phase I clinical study was observed^32^.

The selected binder peptide (denoted as **B9**, Fig. 1) binds with high affinity to the integrin α3 chain and was identified by one-bead-one-compound (OBOC) screening^33^. The eight-residue binder consists of several non-proteinogenic amino acids and has been evolved as a peptidomimetic, cyclized via two terminal cysteine residues, using the one-bead-one-compound approach^34^.

Here, we describe the synthesis and activity of a prototypical ISEr abbreviated **Y9** (Y denotes the trimeric functionality of the molecule) that targets specific cell surface structures (here the integrin α_3_ chain) as well as engage the innate immune system (with an N-formyl effector). In a proof-of-concept study, this ISEr effectively prevented tumor formation in an allogenic adenocarcinoma model in guinea pigs.

## Results

### Effector activation of the immune system

**Figure 2:**
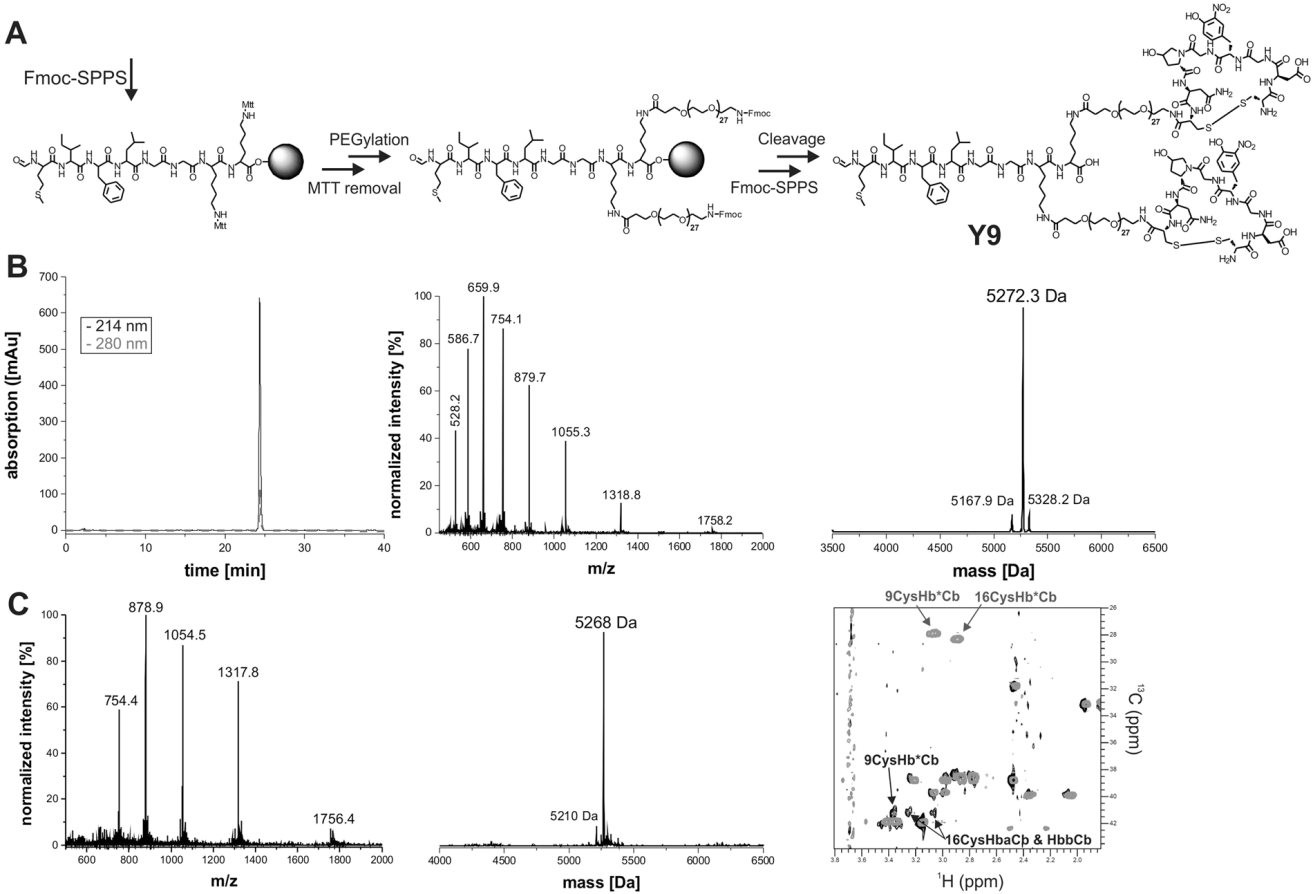
Synthesis and analytical data for **Y9** (**A**) SPPS of **Y9** comprising the fMIFL effector (**F2**) and two integrin α3β1 binder moieties (**B9**). (**B**) Analytical data of purified **Y9** (RP-HPLC, ESI-MS and de-convoluted mass spectrum, calculated MW of fully reduced **Y9** is 5271.0 Da). (**C**) **Y9** was used for all *in vitro* and *in vivo* experiments in its oxidized, disulfide cyclized form as confirmed by ESI-MS (+de-convoluted spectrum, calculated MW of disulfide-cyclized **Y9** is 5268 Da) and NMR experiments. These experiments confirmed the correct assembly of **Y9** and formation of both disulfide bridges. Formation of the disulfide bond between Cys 1 and Cys 8 in the binder peptide of **Y9** results in a change in the chemical shifts of the Cys Cβ resonances. An overlay of the ^1^H-^13^C HSQC NMR spectra of reduced (grey contours) and oxidized (black contours) **Y9** is shown with Cys Cβ peaks labeled. Additional information on chemical shifts can be found in Table S2.

**Figure 3:**
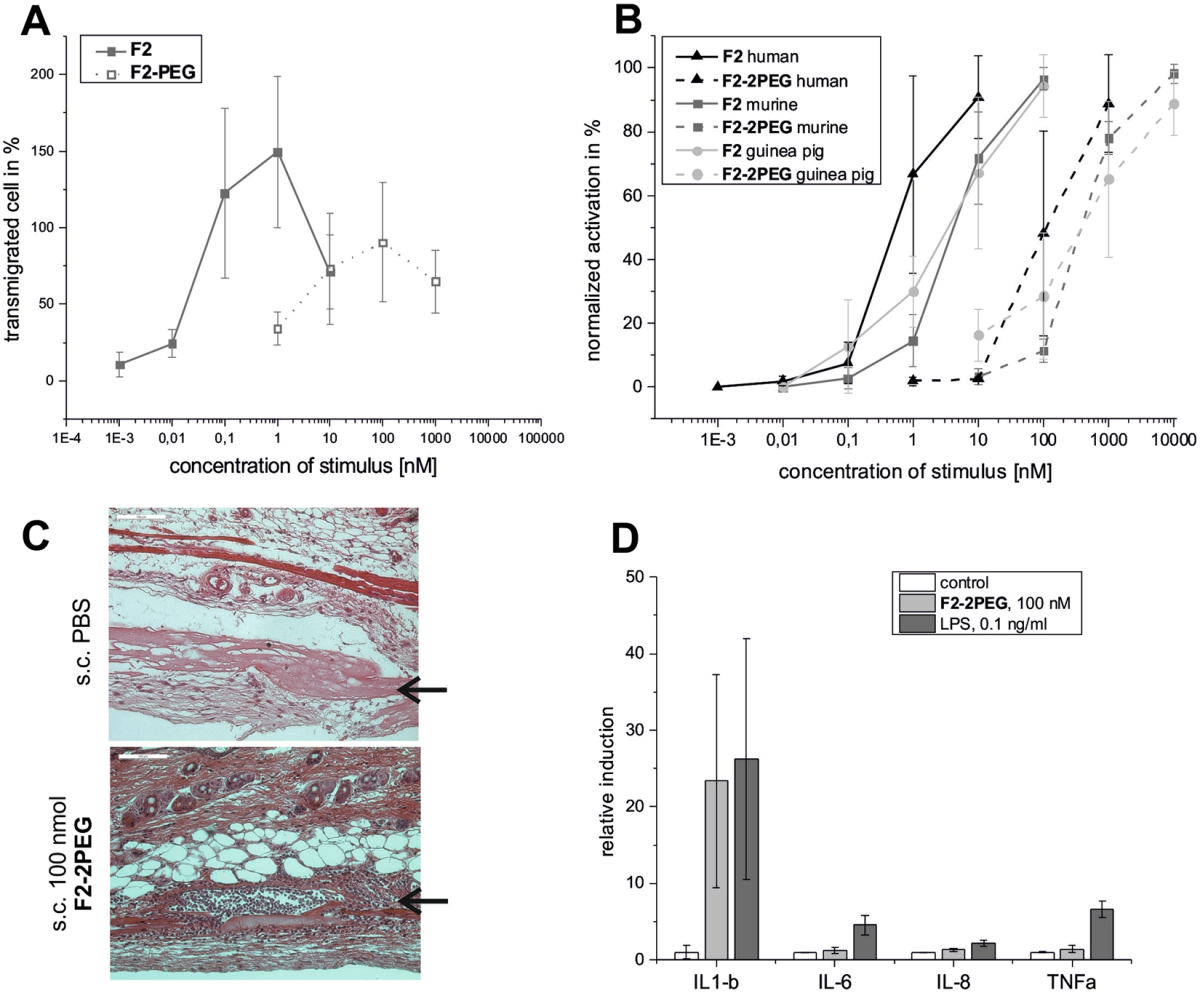
Analysis of effector **F2**. (**A**) Chemotaxis of human leukocytes upon stimulation with different concentrations of (PEGylated) effector. (**B**) Oxidative burst of human, murine and guinea pig leukocytes upon stimulation with (PEGylated) effector. In panels A and B, data of three independent experiments are averaged. (**C**) Immune cell infiltration in mouse skin (HE-stained sections) 24 h after subcutaneous injection of PBS buffer (top) and 100 nmol **F2-2PEG** (bottom) into mouse skin. Infiltrating immune cells are visible as small dark dots (scale bar 100 µm). Black arrows indicate matrigel depots under the skin. (**D**) Cytokine release from human monocytes upon stimulation with **F2-2PEG** or LPS (averages of two independent experiments with monocytes from different donors).

Separate testing of the two components comprising the ISEr **Y9**, the effector (**F2**) and the binder moiety (**B9**), were necessary to select suitable building blocks as well as to learn what changes of the individual properties occur upon incorporation into an ISEr (Figs 1A and 2A). We initially started with testing the well-known N-formyl peptide effector fMLF (denoted as **F1**, Figure S1) at concentrations between 1–10 nM. fMLF was selected based on prior reports of its use for inducing chemotaxis and immune reactions when linked to antibodies^30,32^. Our experiments confirmed that this short, N-formyl peptide prompts chemotaxis of human leukocytes and superoxide production in human granulocytes (dihydro-rhodamine (DHR) oxidation assay, Figure S2A,B)^35^. However, incorporation of **F1** into a suitable scaffold to attach two binder moieties, consisting of a short glycine-glycine linker and two additional lysine residues linked to PEG_27_ via their ε-amino groups (**F1-2PEG**, Figure S1), reduced the potency to induce chemotaxis and neutrophil activation by two orders of magnitude (Figure S2A,B). This behavior is best explained by the much slower diffusion of the larger molecule and potential occlusion of **F1** by the PEG chains. Additional assays indicated that **F1** and **F1-2PEG** are less potent immune activators in mice and guinea pigs (Figure S2C,D)^36^. In order to incorporate an effector suitable for activation of innate immune responses in humans, mice and guinea pigs, we selected fMIFL (F2), a sequence previously identified from *Staphylococcus aureus* ^37–39^. **F2** induces chemotaxis and superoxide production of human neutrophils starting at 10 pM concentration (Fig. 2A,B). Here, we also observe a loss of potency by 1–2 orders of magnitude when linked to the PEG chains (**F2-2PEG**, Figs 2A,B, S3). However, sufficiently high concentrations of effector to induce an effective immune response could still be reached, as demonstrated by subcutaneous application of **F2-2PEG** in mice and guinea pigs. Analysis of tissue sections of mouse skin samples 24 h after subcutaneous injection of 100 nmol **F2-2PEG** clearly showed infiltration by immune cells at the injection site in comparison to control injected animals (Fig. 3C). We could demonstrate that 6 h incubation of PBMCs with 100 nM **F2-2PEG** increased the levels of IL-1β secreted from human monocytes (Fig. 3D) similarly to bacterial lipopolysaccharides (LPS). Other cytokine levels such as IL-6, IL-8 and TNFα were not significantly increased by **F2-2PEG** while the positive control used here (LPS), induced increases of these cytokines (Fig. 3D).

### Specific binding to cancer cells

**Figure 4:**
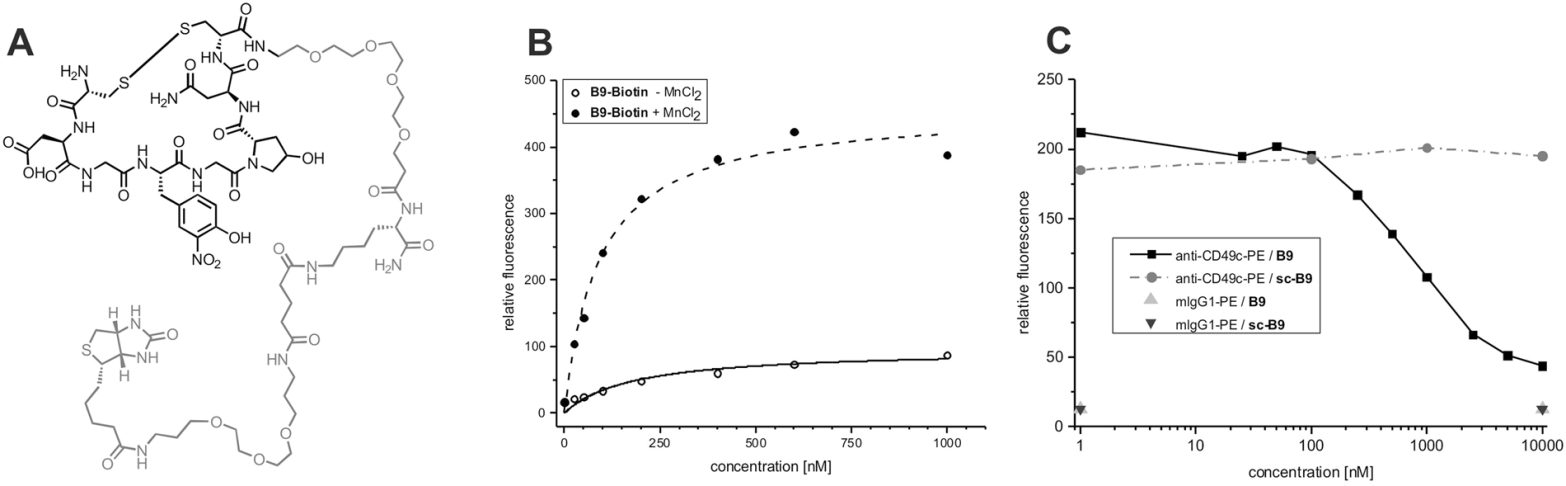
Analysis of binder **B9**. (**A**) Structure of **B9-Biotin**. (**B**) Concentration-dependent binding of **B9-Biotin** to A431 (epidermoid carcinoma cells) expressing integrin α3β1 in the presence and absence of Mn^2+^ to induce the high affinity state of the integrin. Data was fitted based on a one site-specific binding model and gave apparent K_D_ values of 90 nM in the presence of Mn^2+^ and 176 nM in its absence. (**C**) Displacement of an anti-CD49c-PE conjugate by increasing concentrations of **B9**. No displacement of the antibody occurred with scrambled binder (**sc-B9**). An isotype control with mlgG1-PE was used to determine background signal.

Our selected binder **B9** was prepared by Fmoc-based SPPS and carefully analyzed with respect to binding to a panel of human, mouse and guinea pig tumor and blood cells (Tables 1 and S1). For the detection by streptavidin in flow cytometry, **B9** was C-terminally labeled with biotin (Fig. 4A). Concentration-dependent flow cytometry measurements using this **B9** **-Biotin** demonstrated high affinity binding to A431 tumor cells expressing integrin α_3_β_1_ (also known as VLA-3) on their surface (K_D_ ~90 nM, Fig. 3 and S3). **B9** competes with a commercially available anti-integrin α3 antibody (anti-CD49c) for the same binding site, thereby unequivocally establishing the target of this peptide binder (Fig. 4C). Binding affinity depends on the addition of MnCl_2_ (at 2 mM) during incubation as Mn^2+^ induces the high-affinity state of the integrin (Fig. 4B ^40,41^). In the absence of Mn^2+^ the apparent K_D_ drops to 176 nM.

**Table 1:** Flow cytometry based analysis of anti-CD49c, **B9-Biotin** and **Biotin-Y9** binding to various human, mouse and guinea pig cell lines.

**Species**	**Cells and cell lines**	**CD49c**	**B9**	**Y9**
Human	A431	**++**	**++**	**++**
	PC-3	**++**	**++**	**++**
	K562	−	−	−
	HUVEC	**+**	**+**	**++**
	foreskin fibroblasts	nd	−	nd
	primary lymphocytes	−	−	−
	primary monocytes	−	−	−
	primary granulocytes	−	−	−
	primary monocytes 2 days	−	−	nd
	primary macrophages 7 days	**+/−**	**+/−**	**+/−**
Mouse	NIH-3T3	nd	**+**	**+**
	J774A.1	nd	−	nd
	3LL-R	nd	**+**	nd
	Hepa1–6	nd	**+**	nd
	primary hepatocytes	nd	−	nd
	primary leukocytes	nd	nd	−
	primary macrophages 7d	nd	−	−
Guinea pig	GPC-16	**++**	**++**	**++**
	104C1	**++**	**++**	nd
	JH4 clone 1	**++**	**++**	nd

++ Strong binding, + medium binding, +/− weak binding, − no binding, nd not determined.

Additional flow cytometry analyses with a variety of primary cells such as macrophages, fibroblasts and endothelial cells (HUVECs) from humans, mice and guinea pigs were performed to exclude unspecific binding of **B9** (Tables 1 and S1). No binding at concentrations below 1 µM was detected on most of the primary cell types, except for a weak, heterogeneous binding signal observed on human macrophages and HUVEC. Expression of integrin α_3_ on human, rabbit and guinea pig cells was verified with a mouse anti-human CD49c antibody and correlated with binding of **B9** to these cell lines (Table 1). The CD49c specific antibody did not recognize mouse integrin α_3_, however binding of **B9** to most murine tumor cell lines could be demonstrated (Tables 1 and S1).

### Synthesis and characterization of ISEr Y9

The ISEr **Y9** combines **F2** and **B9**, and was synthesized by Fmoc SPPS. N-terminal formylation of MIFLGGKK on resin was quantitatively achieved with *p*-nitrophenylformate^42^ and followed by Mtt removal from both lysine side chains onto which two PEG_27_ linkers were coupled prior to generating identical binder peptides, also via stepwise SPPS. The complete molecule, denoted as **Y9**, was obtained in 20% yield based on the synthesis scale of 0.2 mmol and purified product (207 mg). Purification via RP-HPLC of reduced **Y9** (Fig. 2B) was followed by oxidative cyclization of both binder moieties to give active **Y9** with more than 98% purity (Fig. 2C).

To characterize **Y9** structurally, 2D NMR data were recorded in aqueous solution at pH 3.0 and chemical shifts were assigned for the reduced form of **Y9** (Table S2). Most of the secondary Hα shifts of both the binder and effector peptides in **Y9** were smaller than 0.1 ppm, showing that both peptides are in a predominantly random coil conformation (Figure S5 ^43^). NMR data of the oxidized form of **Y9** were compared with that of reduced **Y9**, confirming (in agreement with MS data) the presence of the disulfide bond. Most notably, the Cβ resonance of the cysteine residues in **B9** shifted from ~28 ppm in reduced to ~41 ppm in oxidized **Y9** (Fig. 2C).

### Activation of the innate immune system by ISEr Y9

**Figure 5:**
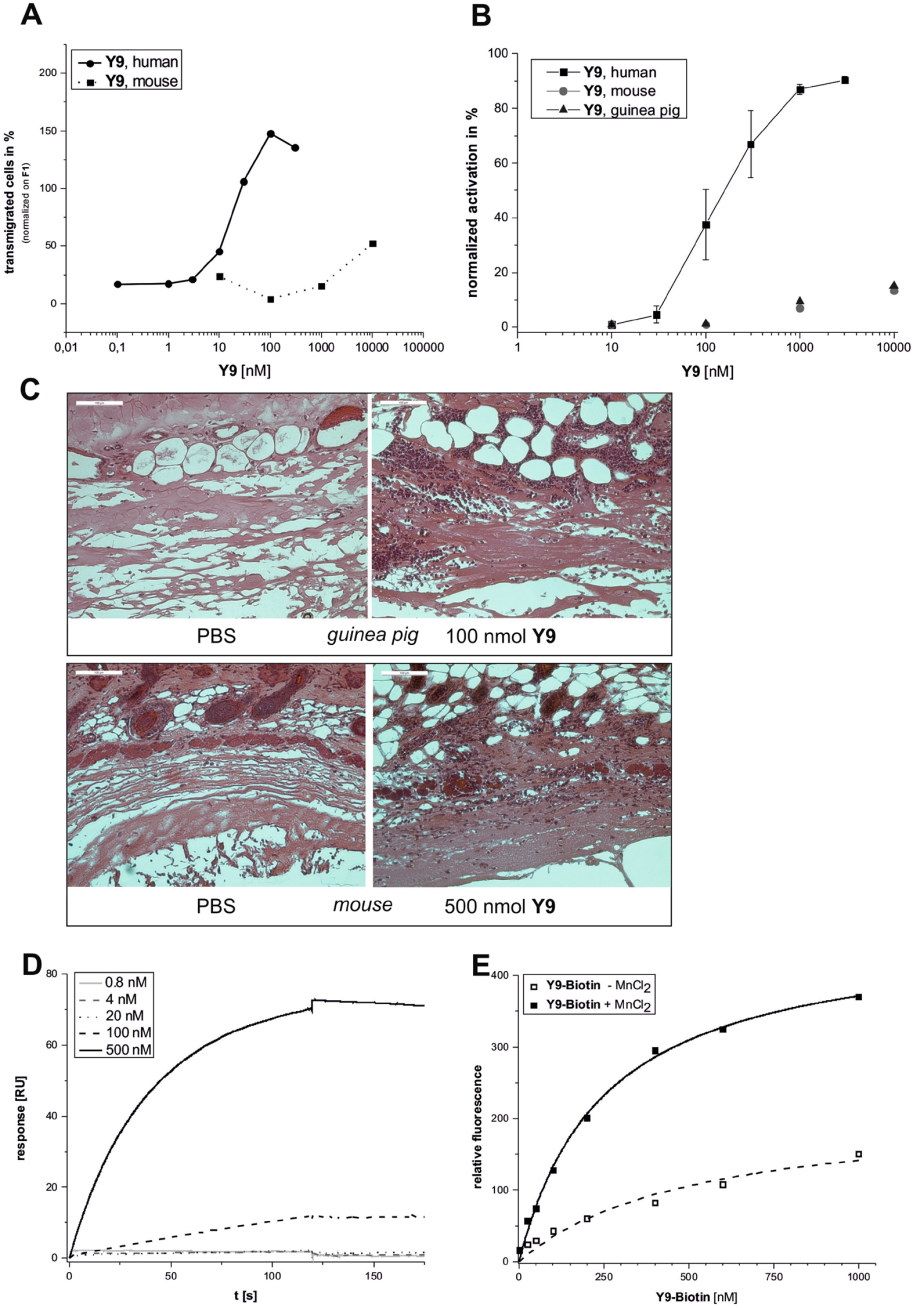
*In vitro* and *in vivo* analysis of **Y9**. (**A**) Chemotaxis of human and murine leukocytes upon stimulation with different concentrations of **Y9**. (**B**) Oxidative burst of human, mouse and guinea pig leukocytes upon stimulation with **Y9**. (**C**) Immune cell infiltration in nude mice and guinea pig skin (HE stained sections, scale bars 100 µm) 24 h after s.c. injection of 100 nmol (guinea pig) or 500 nmol **Y9** (Balb/c^nu/nu^ mice), respectively. (**D**) SPR measurement of **Y9** binding to immobilized integrin α3β1 (VLA-3). (**E**) Concentration-dependent binding of biotinylated **Y9** to A431 cells in the presence or absence of MnCl_2_ (detected by incubation with PerCP-Cy5.5 labeled streptavidin).

**Y9** exhibits similar effector properties to **F2-2PEG** in chemotaxis and oxidative burst assays (Fig. 5A–C). Before using **Y9** in any subsequent experiments we excluded endotoxin contamination using a commercially available test system based on the limulus amebocyte lysate (LAL) assay (Figure S6)^44^. *In vivo* experiments in mice and guinea pigs showed that local immune cell infiltration occurs upon subcutaneous injections (Figs 5C, S7). Immune cell infiltration into the injection site was most pronounced after the application of 100 nmol **Y9** in guinea pigs. Immune infiltrate was also found (but was less intense) after the subcutaneous injection of 100 nmol **Y9** into guinea pigs that were immune suppressed by four weekly injections of cyclophosphamide (CPA) as used in a subsequent efficacy study with the allogenic GPC-16 tumor model in guinea pigs (Figure S7A,B). To explore the possible use of the large variety of xenogeneic tumor models available in mice we also injected **Y9** into immune-deficient Balb/c^nu/nu^ mice. Here, dose-dependent increases in immune infiltrates between 200 nmol to 500 nmol could be observed (Fig. 5C). This finding indicates that higher dosages of **Y9** are required for inducing an immune response in immune-deficient Balb/c^nu/nu^ mice than for immune-suppressed guinea pigs. Immunohistochemical staining of the skin sections with antibodies against the myeloid cell marker myeloperoxidase (MPO) and the macrophage marker F4/80 demonstrated that the immune infiltrate consisted, at least in part, of granulocytes, monocytes and macrophages (Figure S7C–F).

### Specific integrin binding of ISEr Y9

Binding to integrin α_3_ was shown *in vitro* via surface plasmon resonance (SPR) measurement using immobilized integrin α_3_. Here, a K_D_ value of 60 nM for **Y9** was determined (Fig. 5D). This value is in the same range as found for **B9-Biotin** in previous flow cytometry measurements of the binder moiety alone and agrees with measurements of biotinylated **Y9** on cells. To determine binding constants on integrin α3β1 expressing cells, we used biotinylated **Y9** (biotin attached to the N-terminus of the effector) and determined an apparent K_D_ of 244 nM for binding to A431 cells (Fig. 5E). This three-fold decrease in affinity when compared to **B9-Biotin** could be due to an occluding effect of the PEG spacers. To test this hypothesis we used our scaffold (fMIFLGGKK with two PEG_27_ chains) just carrying one **B9** binder peptide. **B9** competed with 200 nM **B9-Biotin** at an IC_50_ of 318 nM whereas the scaffold with only one **B9** binder achieved the same effect only at 1533 nM. This confirmed our hypothesis that the PEG spacers have a negative impact on binding affinity. In competition experiments **Y9** displaced **B9-Biotin** at a four-fold lower concentration than the monovalent binder (380 nM), which also demonstrated the positive effect of bivalency in **Y9** (Figure S8). Further analysis of binding of **B9** and **Y9** under physiological conditions (37 °C) revealed that **Y9** remains present on the surface of various cancer cell lines longer than **B9** (Figure S9). **B9-Biotin** bound to PC-3 or A431 cells disappeared from cells quickly when incubated at 37 °C and no signal above background was detected by streptavidin staining after 5 min. However, biotinylated **Y9** remained on cell membranes with t_1/2_ of at least 15 minutes (Figure S9). To exclude contributions by the effector **F2** to binding of **Y9** to A431 cells, staining with a formyl peptide receptor 1 (FPR-1)-specific antibody confirmed that no FPR-1 was present on these cells (Figure S10).

### Stability and availability of ISEr Y9

The amount of effector available to attract and stimulate immune cells upon administering **Y9** is not only affected by internalization and dissociation but also by its degradation. To this end, we have tested stability of **Y9** in mouse serum using extraction and HPLC-based quantification (Figure S11). **Y9** remains largely intact for extended periods of time (>24 h) by combining the protective properties of the PEG chains against proteolysis as well as by the presence of several non-proteinogenic amino acids in the binder peptides and their cyclic structure (Fig. 2A). After 48 hours only 20% of **Y9** were degraded. Bioavailability of **Y9** *in vivo* was tested in sera taken from mice after subcutaneous application of **Y9**, using a sensitive dot-blot method and a custom-made antibody against **B9** (detection limit ~1 nM, Figure S12). Upon local subcutaneous injection of 200 or 500 nmol of **Y9**, the binder peptide was still detected after 24 h. For injection of 200 nm **Y9** additional measurements of serum concentrations were carried out after 1 h and 7 h showing serum concentrations of **Y9** of 350 and 75 nM, respectively. Based on this data, we estimated a clearance half-life of ~2 hours for **Y9**. In all assays, no major degradation product was found that would point to design weaknesses.

### Effect of ISEr Y9 on tumor formation in guinea pigs

**Figure 6:**
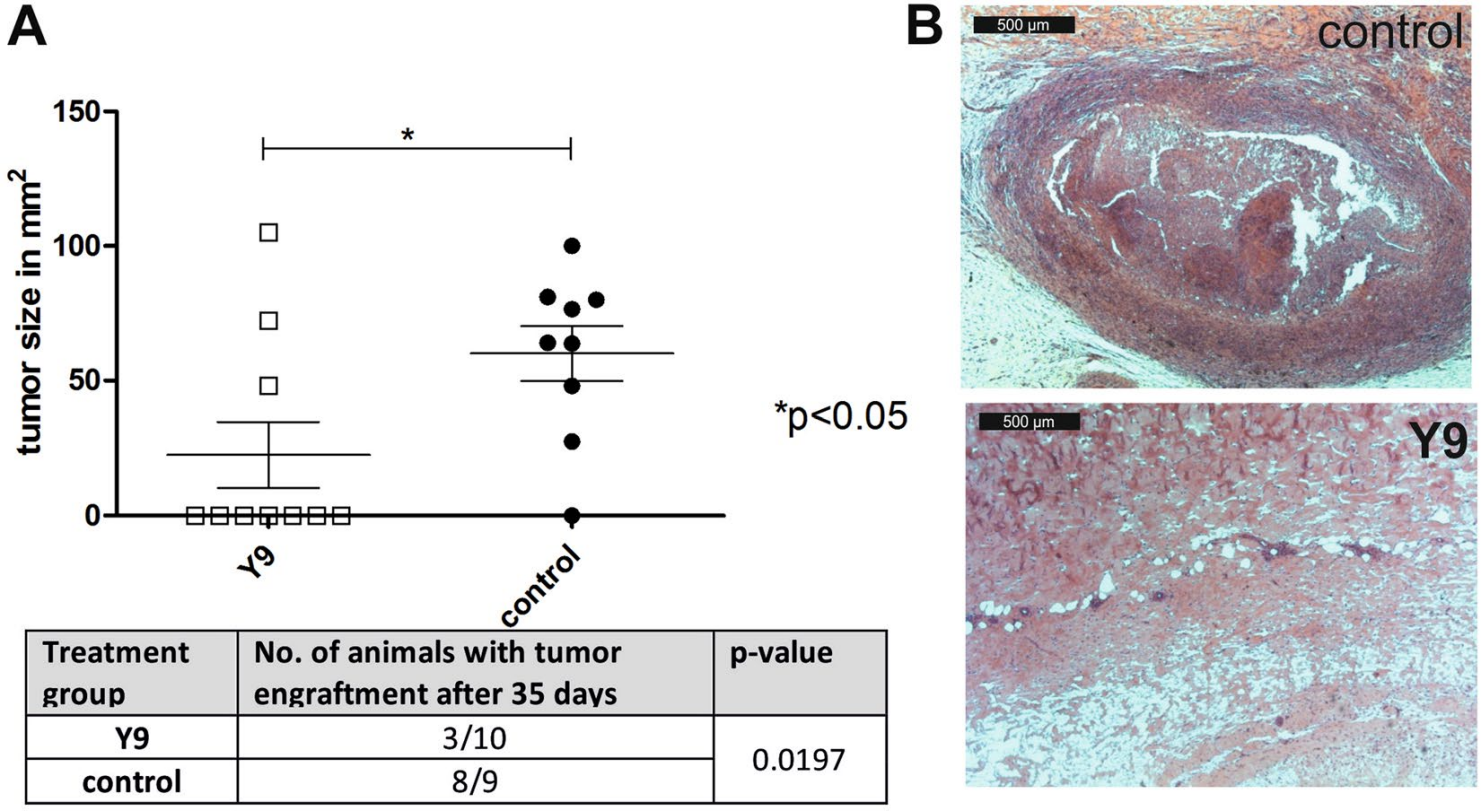
Efficacy of **Y9** tested in a GPC-16 tumor model in guinea pigs (**A**) Measurement of tumor size in GPC-16 inoculated, CPA-treated guinea pigs over 35 days. Animals receiving 1.6–2 × 10^7^ GPC-16 cells mixed with 200 nmol of **Y9** and Matrigel before injection are compared with animals receiving GPC-16 cells premixed only with PBS and Matrigel as a control. With **Y9** treatment only 3 out of 10 guinea pigs carried verifiable tumors whereas in the untreated control group 8 out of 9 animals carried tumors (statistics are based on t-test for the upper graph and on a Wilcoxon-test for the engraftment comparison). (**B**) Tumor growth at day 35 after inoculation in guinea pig skin (HE-sections) treated with PBS control (top) or **Y9** (bottom); 50x magnification.

Antitumor efficacy of **Y9** was demonstrated by administering a single dose of 200 nmol (~1 mg) together with GPC-16 tumor cells subcutaneously into guinea pigs. Based on our *in vitro* data and previous studies indicating successful stimulation of the innate immune system by fMLF-conjugated IgG, guinea pigs are a suitable animal model to test efficacy of **Y9** ^32,45^. We have established a guinea pig tumor model using guinea pig-derived colorectal adenocarcinoma cells (GPC-16^46^) injected subcutaneously into Dunkin Hartley guinea pigs. Since the genetic background of the guinea pig strain from which the GPC-16 tumor originated was unknown and due to the limited availability of specific-pathogen-free (SPF)-guinea pig strains, we established the tumor growth under immunosuppression with cyclophosphamide (CPA)^47^. Final efficacy studies were then performed under CPA treatment (200 mg/kg body weight) one day before tumor inoculation and thereafter once per week for 4 weeks. Two independent series of experiments showed that only in 3 out of 10 **Y9**-treated animals tumors were present after 35 days, whereas in the control group (GPC-16 cells in buffer) 8 out of 9 animals showed solid tumor formation after 35 days (Fig. 6) as verified by macroscopic and microscopic observations. None of the **Y9**-treated guinea pigs showed any undesired effects besides reddening at the injection site.

### Safety of ISEr Y9 in mice

No effect of **Y9**, at concentrations of up to 10 µM, on the viability and proliferation of different cancer cell lines was observed in MTT assays (Figure S13). To further confirm that **Y9** can be safely applied in animals, we performed initial dose escalation studies testing acute toxicity after repeated application of **Y9** in immune-competent Balb/c mice. Five animals per group were subcutaneously injected with 200, 400 or 800 nmol **Y9** every two days for 14 days. A control group of ten mice received 0.9% NaCl. Four days after the last application of **Y9**, blood and serum parameters as well as relative organ weights were measured and tissue samples of organs were analyzed by histopathology. As expected **Y9**-treated animals exhibited local effects such as increased neutrophil and monocyte infiltrates at the injection site and, correlating with this observation, a mild but significant dose-dependent immune infiltration and lymphoid hyperplasia in the draining lymph nodes was found. No histopathological effects were detected in any of the organs tested (liver, kidney, pancreas, spleen, heart, lung, ovaries, brain, stomach, intestine, mesentery) after repeated 200 and 400 nmol **Y9** injections (Figure S14). Only with the highest dose of 800 nmol moderate degenerative changes of the kidney, including distended tubules and protein casts, were detected. Measuring relative organ weights, a slightly enlarged liver weight was observed. However, no histopathological abnormalities or steatosis was detected in the liver (Figure S15). The analysis of serum and blood samples after the application of the highest dose of 800 nm **Y9** revealed slightly but significant increased lipase and creatinine (CREA) levels and significantly increased amylase, triglycerides, cholesterol and blood urea nitrogen (BUN) levels in serum (Figure S15). Hematological analysis including a differential hemogram of the white blood cells showed no significant changes. Overall, these results do not indicate any significant systemic toxic effect at these effective dosages.

## Discussion

Conventional antibodies, fragments thereof and other protein-based binders address a molecular weight range above 25 kDa, in contrast to small molecule drugs that are in the range of 300 to 1000 Da, which leaves the intermediate mass range open for new concepts such as the ISEr here. The trivalent ISEr **Y9** provides a purely synthetic alternative to conventional antibodies and related biological techniques. It offers a versatile synthesis route to medium sized drug molecules (~5 kDa) that combine bivalent, specific target binding with activation of the innate immune system. Target specificity is achieved by attaching two binders (**B9**) to a generic scaffold carrying fMIFL (**F2**) as an effector for activating formyl peptide receptors (FPRs). This effector induces chemotaxis and activation of human and guinea pig leukocytes *in vitro* and *in vivo* without undesired side effects.

Formyl-peptides can also induce the expression of IL-1β, TNFα and IL-8^48^, but we did not observe a significant induction of the latter two cytokines by **F2**. Our data thus indicates that treatment of tumors with **F2-2PEG** does not contribute to the expression of these potentially tumor-promoting cytokines^49^. Furthermore, the flexible synthesis route allows for the adaption of effectors to specific requirements with respect to immune stimulation and species.

The design of **Y9** allows access to more than one binding site on a cellular target such as in dimeric or clustered receptors, thereby improving binding to the targeted cells beyond simply increasing the local concentration of a receptor ligand towards more complex avidity effects. Such effects will help to accumulate **Y9** at a concentration in tumor tissue sufficient to activate macrophages. Bispecific or multispecific molecules targeting two or more different cell-surface receptors can also be easily envisioned based on this design and synthesis scheme. Such molecules will combine the ease of chemical synthesis with well-known advantages of bi- or multispecific binders, as demonstrated for approved bispecific antibodies such as Catumaxomab^50^. The PEG chains reduced the affinity of **Y9** for integrin α_3_-expressing A431 cells. Nevertheless, efficacy could still be demonstrated *in vivo*, possibly due to the role of PEG in protecting against biodegradation, improving solubility, and promoting the formation of monodisperse molecules in aqueous solution^25^.

**Y9** reduced the establishment of a GPC-16 based tumor in guinea pigs by 67% upon a single dose treatment during injection of tumor cells. As no direct toxic effects of **Y9** on cancer cells were observed *in vitro*, we conclude that activation of immune cells interfered with tumor establishment. Clinical use of such a treatment can be envisioned in preventing tumor relapse or development of metastases after first line treatments such as surgery, chemotherapy and/or radiation. As the simultaneous injection of tumor cells and **Y9** mimics a local administration of the drug, as for instance anticipated during chemoembolization or peri-surgical application, other therapeutic applications are possible as well. Considering that **Y9** was still effective in cyclophosphamide treated guinea pigs, it might provide an effective treatment in combination therapies. No severe toxic effects other than the expected moderate local reactions to **Y9** were observed in guinea pigs and mice. Only repetitive doses four-fold higher than used in the effective treatment led to mild pathological changes in kidneys and some serum and blood parameters. The moderate degenerative changes of the kidney including distended tubules and protein casts in the highest dosage group indicate renal clearance of **Y9**. In general, **Y9** can be safely used within a dose range of 100 to 400 nmol (0.5–2 mg).

Overall, **Y9** is an alternative to conventional antibody-based therapeutics and related protein-based approaches due to its entirely flexible and robust chemical synthesis. ISErs can be generated quickly, providing access to customized drugs targeting specific receptors not only relevant for cancer therapy but also for other disease areas requiring modulation of innate immune responses at distinct target sites such as inflammatory and autoimmune diseases.

## Online Methods

### Synthesis of effector and binder peptides

Peptides were obtained via standard Fmoc SPPS. In brief, the first amino acid was DIC-activated and subsequently coupled to Wang resin (100–200 mesh, 0.9 mmol/g substitution level) in the presence of DMAP as a catalyst. All following couplings were performed using HBTU as activator. To check for complete coupling, a Ninhydrin test was conducted. Fmoc was removed using 20% piperidine in DMF. Formylation was achieved using 3 eq *p*-nitrophenylformate in DMF for 3 h. The peptide was cleaved using TFA/TIS/H_2_O/DMS 92.5:2.5:2.5:2.5 (v/v) for 2 h, following peptide precipitation with cold diethyl ether and re-solubilization in ACN/H_2_O 1:1 (v/v) with 0.1% TFA. After freeze drying, peptides were dissolved in 6 M GndHCl, pH 4.7 and purified via RP-HPLC.

### Synthesis of Y9

Removal of the Mtt protecting group was achieved by flow washing the peptidyl-resin with DCM/TFA/TIS 98:1:1 (v/v) until the solution turned colorless. Each lysine side chain was PEGylated overnight using 1.12 equivalents of Fmoc-NH-(PEG)_27_-COOH and HATU as activator. Subsequently, the synthesis was continued using standard SPPS methods as described above.

### Surface plasmon resonance (SPR)

Single cycle kinetic experiments were conducted on a Biacore 3000 system using a CM5 sensor chip at 25 and 37 °C with a flow rate of 10 µl/min. After coupling of the VLA-3 receptor (R&D Systems) via standard EDC/NHS immobilization chemistry (7 min activation with EDC/NHS followed by a 7 min flush with VLA-3) in 10 mM NaOAc buffer at pH 6 containing 1 mM MnCl_2_, a 1 mM solution of ethanolamine was injected for 7 min to block all amine reactive sites. Subsequently the analyte was flushed over the chip surface at a flow rate of 10 µl/min in HEPES buffered saline supplemented with 1 mM MnCl_2_. As no method was found to regenerate the surface from **Y9** without damaging the VLA-3, **Y9** was injected in increasing concentrations, taking care not to reach saturated binding (single cycle kinetics).

### Animals

All experiments were conducted in accordance with the German legislation governing animal studies. The Principles of Laboratory Animal Care (Guide for the Care and Use of Laboratory Animals: Eighth Edition. Washington, DC: The National Academies Press, 2011) were followed. The animal protocol was approved by the Governmental Animal Care and Use Committee (LANUV AZ. 87-51.04.2010.A278). All experiments were performed in the Institute for Laboratory Animal Science, a DIN ISO 9001/2008 certified facility.

### Human cells

Primary cells were obtained from healthy volunteers that served as donors at our blood bank, after written informed consent and approval by the local ethics committee of the Uniklinik RWTH Aachen, Germany.

For more information on experimental details about NMR measurements, binding and stability assays as well as on animal testing, please see Supplementary information.

## Electronic supplementary material

Supplementary Information
