# Supplementary material for: Synthetic integrin-binding immune stimulators target cancer cells and prevent tumor formation
Source: Sci Rep. 2017 Dec 14;7:17592. doi: 10.1038/s41598-017-17627-0 (PMC5730604; doi:10.1038/s41598-017-17627-0)
Supplement: Supplementary file 1 — Supplementary Information [file 41598_2017_17627_MOESM1_ESM.pdf]

Supplementary Information

Synthetic integrin-binding immune stimulators target cancer cells and prevent tumor formation

Manuel Brehs[1], André J.G. Pötgens[2], Julia Steitz[3], Karine Thewes[1], Janett Schwarz[2], Anne C. Conibear[1], Matthias Bartneck[4], Frank Tacke[4], Christian F.W. Becker[1]*

Materials and Methods

9-Fluorenylmethoxycarbonyl (Fmoc)-protected amino acids, 2-(1H-benzotriazol-1-yl)-1,1,3,3-tetramethyluronium hexafluorophosphate (HBTU), and Wang resin were purchased from Novabiochem. Trifluoroacetic acid was from Roth (Karlsruhe, Germany). Fmoc-PEG$_{27}$-COOH was from Polypure (Norway). All other chemicals were obtained from Sigma-Aldrich (Vienna, Austria) at the highest purity available and used without any further purification.

*Binding of **B9** and **Y9** to cells*

Binding of **B9** or **Y9** to cells was determined using flow cytometry. Cell lines were acquired from DSMZ or LGC and cultured according to the distributors' guidelines. Human, murine or guinea pig leukocytes or macrophages were isolated and cultured as described below. Cells growing in monolayers were detached using trypsin/EDTA and then re-suspended in complete culture medium. After washing with washing buffer (WB: 0.9% NaCl, 10 mM Hepes, pH 7.3) at 4°C, cells were re-suspended at 2x10^6 cells/ml in peptide blocking buffer (PBB: 0.9% NaCl, 10 mM Hepes, pH 7.3, 5 mM KCl, 3% BSA, 5% FCS) and incubated for 10 minutes on ice before the addition of **B9** or **Y9**. Biotinylated **B9** or **Y9**, diluted to twice the intended concentration in PBB were added to equal volumes of aliquots of 1x10^5 cells, and were incubated for 30 min on ice with repeated shaking to keep the cells in suspension. Except where indicated, 2 mM $MnCl_2$ was added to incubations with **B9** or **Y9**. Cells were washed three times with 1 ml WB, followed by incubation with streptavidin labeled with PerCP-Cy5.5 (BD Biosciences, 200 µg/ml; diluted 1:400 in PBB) for 15 min on ice. After one additional washing step with phosphate-buffered saline (PBS) at 4°C, samples were re-suspended in PBS with Hoechst 33258 solution (0.5 µg/ml) and analyzed using a FACS Canto system (at least 1x10^4 cells per sample were counted). Evaluations were performed using FloJo software. Whole cells were gated in a FSC/SSC plot and live gating was based on Hoechst exclusion. After gating out debris, aggregates and dead cells, overlay histograms in the PerCP-Cy5.5 channel were made of cells incubated with **B9**/**Y9** or with control or no peptide at all, and in indicated cases the geometrical mean fluorescence was recorded. Variations on this method:

1. To determine which cells or cell lines bind **B9**/**Y9**, 1 µM of the biotinylated compound was used.
2. For determining binding affinity, a range of concentrations of the biotinylated compound was tested and the fluorescent signals were plotted against concentration. The concentration at which half-maximum binding intensity was reached (K_D) was calculated using Origin 9.0 software and a "one site specific binding" fit ($y=(B_{max}*x)/(K_D+x)$).
3. For determining relative binding affinities of non-biotinylated **Y9**, a fixed concentration of **B9-Biotin** (200 nM) was mixed with increasing concentrations of **Y9** before cells were added. The fluorescent signals were plotted against competitor concentration. The concentration at which

half-maximum inhibition of binding intensity (K_i) was reached was calculated using GraphPad Prism.
4. To test the stability of biotinylated **B9**/**Y9** on the cell membrane, **B9**/**Y9** incubated cells were washed as described above and then re-suspended in Eagle's minimum essential medium (MEM - a cell culture medium lacking biotin) with 10% FCS and 10 mM Hepes, pH 7.3 and incubated on ice or at 37°C. At different time points after washing 50 µl samples were taken and added to 50 µl of Streptavidin-PerCP-Cy5.5 (diluted 1:200), incubated on ice for 15 min, washed with PBS, and analyzed in PBS with Hoechst solution. Fluorescent signals were plotted against time between washing and streptavidin addition.
5. To study competition of binding between **B9**/**Y9** and anti-Integrin α3 antibody, cells were blocked in PBS + 2% human serum, 2% murine serum, 2% rabbit serum and 2 mM $MnCl_2$ and incubated with a mixture of anti-human CD49cPE (R&D systems, FAB1345) diluted 1:400 (final dilution) and different concentrations of **B9**/**Y9** in the same blocking buffer. After an incubation of 30 min on ice, cells were washed with 1 ml PBS and analyzed by flow cytometry (PE channel) in PBS with Hoechst. The fluorescent signals were plotted against competitor concentration.

Effector assays
Isolation of human leukocytes
Leukocytes were isolated from heparinized blood from healthy volunteers. Red cells were sedimented by incubating with dextran 500,000 at 37ºC and the upper layer containing the leukocytes was subjected to two rounds of hypotonic lysis (re-suspension of pellets in PBS, addition of 10 volumes of sterile distilled water and incubation for 20 seconds at RT, followed by the addition of 1/10 volume 10 x PBS) to eliminate residual erythrocytes. Guinea pig leukocytes were also isolated from heparinized blood, using the same method.

Isolation of murine leukocytes
Femurs were dissected from C57BL/6 mice and the bone marrow was flushed with PBS using a 26 gauge needle. Red cells were lysed using BD Pharm lyse (Becton Dickinson) lysis buffer according to the manufacturer's instructions.

Culture of murine macrophages.
Bone marrow derived leukocytes were plated in 10 cm petri dishes in RPMI + 10% FCS mixed with 0.25 volume of supernatant of murine fibroblast cell line L-929. Adherent cells developed into macrophages. Medium was changed every two to three days. Macrophages were used in assays after 7 days of culture, and they were detached from the dishes using a cell scraper.

Isolation of human monocytes.
Monocytes were isolated from Buffy coats from the hospitals' blood transfusion service. Mononuclear cells were isolated using centrifugation on a Ficoll density gradient and the PBMC fraction was isolated and washed at least 4 times with PBS. Mononuclear cells were plated in 24-well plates at 4 million cells/well in RPMI + 10% FCS and incubated at 37ºC and 5% CO_2. After 1 h the non-adherent cells were removed by 3 rounds of washing with RPMI+1% FCS and adherent cells were further cultured overnight at 37ºC and 5% CO_2 in 1 ml RPMI+10% FCS. Monocytes were used for assays the next day.

Chemotaxis assays
Human leukocytes were re-suspended in Hank's Buffered Saline Solution (HBSS, without Ca^{2+}, Mg^{2+}) with 10 mM Hepes pH 7.3 and 0.3% BSA at 10^7 cells/ml. Cells were pre-warmed at 37°C for 20 min. After adding the effector dilutions in the lower compartment of 24-well plates (800 µl/well), a Millicell culture plate insert with 5 µm pore size was placed in each well, followed by immediate addition of 200 µl (2 million) leukocytes. In one well, the cells were added without a filter insert, allowing determination of the input number. The plate was incubated at 37°C and 5% CO_2 for 45 min. 5 mM EDTA solution was added and the plate was incubated for another 10 min at 37ºC. The total number of leukocytes that migrated to the bottom chamber was determined using a Neubauer chamber, or the numbers of transmigrated monocytes, granulocytes and lymphocytes were determined by flow cytometry (discriminating cell populations in FCS/SSC plot). Murine macrophages were scraped from the culture plates and added into the filter insert ($1x10^6$ cells in 0.2 ml medium). The plate was incubated for 4 h at 37°C and 5% CO_2. Macrophages on the bottom side of the filter were counted. To this end, the cells at the top side were removed using Q-tips, and the filters were fixed in 4% formaldehyde, washed three times with PBS, incubated 15 min in PBS + 0.5% Triton X-100 + 1% FCS, washed three times with PBS and once with distilled water. The filters were cut out of the inserts and immersed in a drop of mounting media containing DAPI on a slide and sealed under a coverslip. Photographs were taken of ten microscopic fields of each filter using a fluorescence microscope in the DAPI channel. Counting of nuclei was performed using Image J software, means and standard deviations were calculated and presented as absolute numbers (cells/microscopic field) or as fold induction compared to the unstimulated situation.

Oxidative burst assay
Activation of the oxidative burst (phagocyte NADPH oxidase) was measured using a dihydrorhodamine (DHR, Molecular Probes) oxidation assay, based on a method described previously[1]. In brief, 200 µl samples of human, murine or guinea pig leukocytes ($1.25x10^6$ cells/ml in HHBS + 10 mM Hepes, pH 7.3, 0.3% BSA and 5 mM EDTA) were loaded with DHR and catalase (Sigma-Aldrich) and cytochalasin B (Sigma-Aldrich) were added. Cells were pre-incubated at 37°C for 10 min followed by the addition of 50 µl of effector dilutions (5x the intended concentration) in the same buffer and another 15 min of incubation at 37°C. Phorbol 12-myristoyl 13-acetate (PMA, 3 µg/ml) was used as a positive control. All samples were fixed in 1% formaldehyde Hoechst 33258 solution (0.5 µg/ml) was added and cells were kept on ice until analysis using a FACS Canto system. After gating for live cells and granulocytes (human cells) or non-lymphocytes (murine cells) in the FSC/SSC plot, rhodamine-positive cells were identified in a PE/SSC plot, using an unstimulated sample as a reference.

Cytokine release assay
Human monocytes, after an overnight pre-culture, were incubated for 6 more hours in 1 ml RPMI+10% FCS containing 100 nM F2-2PEG, 0.1 ng/ml LPS (Sigma-Aldrich), or no stimulus. All experiments were performed in duplicates. Supernatants were centrifuged and aliquots of 150 µl were stored at -80ºC until cytokine levels were measured with commercially available ELISA kits (Ready-set-go system, eBiosciences). Cytokine concentrations in supernatants were measured and calculated based on the standards included within the ELISA kits and as described by the manufacturer's instructions. The following kits were used: Human TNFα (88-7346), human IL-1β (88-7010), human IL-6 (88-7066), human IL-8 (88-7086). When the samples showed cytokine levels higher than the calibration range, samples were diluted before analysis. Measured cytokine levels were normalized to 1 (the average of the two unstimulated samples).

Serum stability assay

Y9 was added to freshly harvested mouse serum (final concentration 60 µM). After gentle vortexing the sample was incubated for 48 h. At specific time points, the solution was again vortexed, a sample (95 µl) was withdrawn and subjected to ultrafiltration (Vivaspin 500, 10 kDa cutoff; 15 min, 16,000 g). The filtrate was put on ice while the filter remainder was mixed with an equal volume of ethanol (95%), vortexed and centrifuged (5 min, 16.000 g). With the resulting supernatant, this procedure was repeated. The final supernatant was mixed with an equal volume of filtrate, shock frozen in liquid nitrogen and stored at -80 °C until analysis. RP-HPLC analysis was performed using an analytical C4-RP column with a gradient of 5-65% acetonitrile (ACN) in water (containing 0.1% trifluoroacetic acid (TFA)) and detecting **Y9** via absorption at 214 and 375 nm (specific absorption maximum of the nitro-tyrosine building block in **Y9**). **Y9** retention time was verified using pure peptide. The absorption peak observed at the specific retention time of **Y9** was integrated using Origin 9.0 software.

Animals

All experiments were conducted in accordance with the German legislation governing animal studies. The Principles of Laboratory Animal Care (Guide for the Care and Use of Laboratory Animals: Eighth Edition. Washington, DC: The National Academies Press, 2011) were followed. The animal protocol was approved by the Governmental Animal Care and Use Committee (LANUV AZ. 87-51.04.2010.A278). All experiments were performed in the Institute for Laboratory Animal Science, a DIN ISO 9001/2008 certified facility. 6-8 week old C57Bl/6/N, Balb/c, Balb/c$^{nu/nu}$ and 8-10 week old female Dunkin Hartley guinea pigs from Charles River GmbH (Sulzfeld, Germany) were used. Mice were housed in individual ventilated cages under SPF-conditions according to the FELASA guidelines at 22 +/- 1°C, 50 +/- 20% relative air humidity and a 12h dark/light cycle. Water and standard animal species specific diet (Sniff, Soest, Germany) was given *ad libitum*. Guinea pigs were housed in conventional open cages in the same way as describe for mice. Animals were allowed to acclimatize to their surroundings for at least one week before procedures were performed.

Assessment of effector functions in a local skin inflammation model

For studying the local effects of a compound, Dunkin Hartley guinea pigs, Balb/c or Balb/c$^{nu/nu}$ mice were subcutaneously injected with effector peptide **(F1, F2), Y9** or buffer only mixed with Matrigel (BD Matrigel Basement Membrane Matrix, growth factor reduced, #356230, BD Bioscience). After 24 h the animals were sacrificed by isoflurane overdose and injection sites were resected, fixed in methacarn (60% absolute methanol, 30% chloroform, 10% acetic acid), paraffin embedded, and 3µm sections were stained with hematoxylin and eosin. Histological evaluation of the skin sections was performed by a board certified veterinarian pathologist who scored the severity of immune infiltrates (in and around the Matrigel). In mouse skin sections additional immunohistochemical stainings with antibodies against granulocytes/monocytes (anti-MPO) and against macrophages/monocytes (anti-F4/80) were performed. For the MPO-staining sections were incubated for 1 h at 37°C after antigen retrieval with citrate buffer (pH 6) and microwaving (3 x 8 min, 600 Watt) and after blocking with normal goat serum (DAKO, X0907) with a 1:50 dilution of the primary antibody (rat anti-MPO, Abcam, #ab9535). Sections were washed and incubated with a 1:500 dilution of the secondary antibody (biotinylated goat-anti-rabbit Ig, DAKO, #E0432) followed by the color development using the Vectastain ABC (Vector,# PK-6100) and DAB substrate kit (Sigma-Aldrich, #D5905).

For F4/80 staining, skin sections were incubated after blocking with normal goat serum with a 1:500 dilution of the primary antibody (rat anti-F4/80, Abcam, #ab6640) for 1 h at 37°C followed

by incubation with a 1:50 dilution of the secondary antibody (biotinylated goat-anti-rat IgG, BD Pharmingen, #554014). After incubation with horseradish peroxidase (HRP) coupled streptavidin color development was performed using the DAB substrate kit. All immunohistochemical stained tissue sections were counterstained using haemalaun or hematoxylin.

Assessment of anti-tumor efficacy in the GPC-16 tumor model in guinea pig

GPC-16 cells (guinea pig colorectal adenocarcinoma) were acquired from LGC and were cultured in Eagle's minimum essential medium with 10% FCS and 100 U/ml Penicillin and 100 μg/ml Streptomycin. For each experiment 10 female guinea pigs (8-10 weeks old) per group were immunosuppressed with an intraperitoneal injection of 200 mg/kg cyclophosphamide (CPA) one day before the tumor inoculation and repeated every 7-10 days for 4 weeks. Efficacy of the CPA treatment was verified by routine blood analysis before first CPA treatment and on a weekly basis thereafter. For the evaluation of the anti-tumor efficacy of the **Y9** the 10 animals were divided into two groups of 5 animals. 1.6-2 x 10^7 GPC-16 cells re-suspended in Matrigel (BD Matrigel Basement Membrane Matrix, #356234) were mixed with 200 nmol **Y9** or PBS as control and mixtures of 200 µl were subcutaneously injected into the flank of the animals. Body weight and tumor size measurements using a caliper were performed twice a week. 34-35 days after tumor inoculation the animals were euthanized via isoflurane overdose and final blood samples as well as organs (kidney, liver, spleen, lymph nodes) including the injection site/tumor were taken. Organs and tissues were fixed in methacarn, paraffin embedded and 3 μm sections were stained with hematoxylin and eosin for histopathology. The presence of tumors in the skin sections was evaluated by a board certified veterinarian pathologist using a scoring system. Scores of 1= no tumor, 2= unclear diagnosis, 3= rest tumor and 4= solid tumor were used.

Dose escalation study and evaluation of acute toxicity in mice

To study whether a repeated subcutaneous dosing of **Y9** has any local or systemic toxic effects on animals, the following dose escalation study was performed. Groups of 5 Balb/c mice were inoculated subcutaneously with 100 µl of 0.9 % saline (NaCl) solution containing either 200 nmol, 400 nmol or 800 nmol **Y9**. Two control groups (a total of 10 mice) received only 0.9% saline. The injections were repeated every other day for a total of seven injections. Body weight and general health observations were evaluated on a daily basis. At day 4 after the last inoculation of the solutions blood was collected under isoflurane anesthesia for hematology and for serum analysis by retro-orbital puncture. Organs (skin, brain, heart, kidney, liver, lung, ovaries/testis, spleen, local and distal lymph nodes, mesentery, small intestine, stomach, pancreas) were isolated after euthanasia with an isoflurane overdose for histopathology and body weight-normalized organ weights (brain, kidney, heart, liver, spleen) were calculated. Weights of the carcass, brain, heart, kidney, liver and spleen were determined and body weight-normalized organ weight in % was calculated as follows:

$$\text{Body weight-normalized organ weight [\%]} = \frac{\text{organ weight [g]}}{\text{carcass weight [g]}} \times 100$$

The following serum parameters were analyzed with the Vitros 250 (Johnson&Johnson) machine measuring parameters at 25°C: Creatinine Kinase (CK), alkaline Phosphatase (ALP), Amylase, GOT/AST, GPT/ALT, GGT, LDH, Lipase, total protein (TP), Glucose (GLU), Cholesterol (CHOL), Triglyceride (TRIG), blood urea nitrogen (BUN), Creatinine (CREA), total bilirubin (TBIL), inorganic Phosphorus (PHOS), Sodium (Na), Potassium (K), Calcium (Ca),

Albumin (ALB). In EDTA-blood samples and in blood smears the following parameters were analyzed using the celltac α (MEK-6450K, Nihon Khoden) machine and a standard microscope for further differentiation of the white blood cell population: white blood cells (WBC), red blood cells (RBC), platelets (PLT), hemoglobin (HGB), hematocrit (HCT), Mean corpuscular volume (MCV), Mean corpuscular hemoglobin (MCH), Mean corpuscular hemoglobin concentration (MCHC), banded neutrophils, segmented neutrophils, lymphocytes (Lymphs), monocytes (Monos), eosinophils (EOS), basophils (BASO). The organ sections were histopathological evaluated by a board certified veterinarian pathologist who scored the severity of any pathological abnormalities found. Scores: 1: non or negligible, 2: mild, 3: moderate, 4: severe pathological changes.

*Determination of **Y9** levels in animal serum*

With the purpose of developing sensitive methods for detection of **Y9** in biological fluids and/or in histological sections, a polyclonal antibody was generated against **B9**. Two rabbits were immunized with **B9** at Eurogentec (Seraing, Belgium) according to standard protocols (https://secure.eurogentec.com/product/research-anti-peptide-87-day-polyclonal-packages.html?country=nld; AS-PCAP-Rabbit program). The antiserum demonstrating the highest titer towards **B9** in an ELISA assay was affinity purified on a **B9**-coated column, also according to standard procedures. From 20 ml antiserum, 1.5 mg purified antibody were isolated and stored in aliquots at -20ºC in PBS + 0.01% thimerosal, 0.1% BSA diluted 1:1 with glycerin (final concentration 227 µg/ml). For detection of **Y9** in serum of mice taken 24 h after subcutaneous injection with 200 or 500 nmol **Y9**, 1 µl serum samples were spotted in triplicate on a nitrocellulose membrane. For calibration, control serum was spiked with **Y9** in concentrations between 0.5 nM and 20 nM and samples were also spotted in triplicates. After drying, the membrane was soaked in TBST (150 mM NaCl, 50 mM Tris-HCl, pH 7.4, 0.05% tween), blocked for 1 h at RT with 5% nonfat dry milk in TBST. The membrane was washed 3 times in TBST, incubated overnight at 4ºC with the affinity purified anti-**B9** antibody diluted 1:2000 in TBST, washed 3 times, incubated 1 h at RT with anti-rabbit-HRP (GE Healthcare) diluted 1:5,000 in 5% nonfat dry milk in TBST. The membrane was finally washed 3 times in TBST and then incubated with ECL prime substrate (GE Healthcare). Chemiluminescent signals were recorded in an LAS-4000 mini Image reader (Fuji). Spot intensities were quantitated using Image J software. The calibration samples were used to prepare a linear regression curve. The **Y9** concentrations in the mouse sera were determined using linear regression.

Statistical analysis

For the statistical analysis, mean (M) and standard deviation (SD) were calculated. The statistical analysis was performed with the GraphPad Prism Software, Version 5.0 (Graph Pad, Vermont, USA). For the dose escalation and acute toxicity study, significance between groups was evaluated by one-way ANOVA and Dunnett's Multiple Comparison post-test. In case of relative organ weight measurements two-way ANOVA and Bonferroni post-test was used. p-values of < 0.05 were considered significant. In the figures, p-values of < 0.05; < 0.01 and < 0.001 are indicated with one, two or three asterisks, respectively.

NMR measurements

Chemical shifts were assigned from NMR spectra (600 MHz, Varian) recorded on synthetic **Y9** in reduced form (MW 5271.0; 3.5 mg) dissolved in 90% H_2O/10% D_2O (500 µL) at pH 3.0. Two-dimensional NMR spectra (^1H-^1H TOCSY, ^1H-^1H NOESY, ^1H-^{13}C HSQC and ^1H-^15N HSQC)

were recorded at 278 K and referenced to the water signal. Spectra were assigned with CCPNMR[1] using the sequential assignment protocol. Secondary Hα chemical shifts were calculated as the difference between the measured Hα chemical shift and the respective random coil shift.[2] Random coil shifts were temperature corrected using the correction factors calculated by Kjaergaard et al.[3] For the D-amino acids, the random coil shift of the respective L-amino acid was used, the random coil shift of Tyr was used for Tyr(3-NO2) and 4.54 ppm was used for Hyp Hα . Oxidized **Y9** (MW 5268.0) was prepared by dissolving **Y9** in Na_2HPO_4 buffer (50 mM, pH 7.0). After HPLC purification, the oxidized **Y9** (2.1 mg) was dissolved in 90% H_2O/10% D_2O (500 µL) at pH 2.9 and NMR spectra were acquired as described above.
The NMR data showed reasonable dispersion, however a large signal at 3.66 ppm from the methylene protons of the two PEG_{27} chains made phasing difficult and obscured some NOE signals.

Staining of FPR-1 on human leukocyte subpopulations and tumor cell lines
The main receptor for effector peptide **F2** is FPR-1. A FITC-conjugated monoclonal antibody (R&D FAB3744F, mIgG2a) was incubated with human tumor cells as well as with primary human leukocytes. Cells (100,000 cells of the tumor cell lines and 500,000 leukocytes in 100 µl volumes) were incubated for 30 min on ice in blocking buffer (PBS with 2% bovine serum albumin, 2% human serum, 2% mouse serum, 2% rat serum, BSA and sera acquired from Sigma Aldrich) with the antibody diluted 1:50. Control antibody was a FITC-conjugated isotype control (mIgG2a-FITC, R&D IC003F) also diluted 1:50. Unbound antibody was removed by adding 1 ml PBS, pelleting of the cells, and resuspension in 100 µl PBS with Hoechst dye. Stained cells were analyzed by flow cytometry.
Cell lines were gated for single and live cells as explained elsewhere. Leukocytes were gated for live cells in an FSC/Hoechst plot, and then into three subpopulations: granulocytes, monocytes and lymphocytes, as shown in figure S13. Staining of the various cell types is shown in overlay histograms.

*MTT assay to test the sensitivity of tumor cell lines to **Y9***
To test whether the interaction of Y9 with the integrin receptor on tumor cell lines, or an interaction of its effector peptide with a formyl peptide receptor that might be present on some tumor cell lines has an influence on the viability or the proliferative potential of these cells, MTT assays were performed. A431, U-87 or HT-29 cells (all of them positive for integrin $\alpha_3\beta_1$ and all of them binding **Y9** to a high level) were plated in 96-well plates. For 24h assays, cells were plated in 50 µl volumes at 20 000 cells per well in the respective culture medium (RMPI or EMEM) with 5% FCS. For 72h assays cells were plated at 3000 cells per well in medium with 1% FCS. After 2h of adherence, **Y9** or a variant (**sc-Y9**) with scrambled binding peptides (sc-B9) and a functional effector peptide (F2) were added in 50 µl volumes in the same media to final concentrations between 100 nM and 10 µM. All concentrations were tested in triplicates. At the end of the incubation period (24 h or 72 h at 37°C in 5% CO2) cells were 30-80% confluent. MTT reagent (Cell growth determination kit, MTT based, Sigma Aldrich) was added (10 µl per well) and incubation was continued for 2 h. Finally 20 µl 3% SDS as well as 80 µl isopropanol with 0.04M HCl were added and absorbances were measured at 595 nm. Absorbances were plotted against the agent's concentrations. A high absorbance value indicates that a large fraction of MTT was reduced to formazan and that more living cells are present in the well as compared to wells with low absorbance values.

Table S1: Flow cytometry based analysis of anti-CD49c, **B9-Biotin** and **Biotin-Y9** binding to various cell lines

Species	Cell line	CD49c	B9	Y9	Species	Cell line	CD49c	B9	Y9
Human	A431	++	++	++	Mouse	NIH-3T3	nd	+	+
	U-87MG	++	++	++		L-929	nd	-	-
	U-138MG	++	++			MHEC5-T	nd	+	
	PC-3	++	++	++		J774A.1	nd	-	nd
	K562	-	-	-		3LL-R	nd	+	nd
	MCF-7	+	+			3LL-S	nd	+	nd
	MDA-MB-231	++	++	++		MC38	nd	-	nd
	Caco-2	nd	-	-		EG7	nd	-	nd
	HT-29	+	++	++		E0771	nd	-	+
	A549	nd	++			TSA	nd	+	nd
	HeLa	nd	++	++		4T1	nd	+	++
	HMC-1	nd	nd	-		C26	nd	+	nd
	HUVEC	+	+	++		Hepa1-6	nd	+	nd
	foreskin fibroblasts	nd	-	nd		primary hepatocytes	nd	-	nd
	fresh lymphocytes	-	-	-		leukocytes	nd	nd	-
	fresh monocytes	-	-	-		macrophages 7d	-	-	-
	fresh granulocytes	-	-	-	Guinea pig	GPC-16	++	++	++
	monocytes 2 days	-	-			104C1	++	++	nd
	macrophages 7 days	+/-	+/-	+/-		JH4 clone 1	++	++	nd
					Rabbit	VX2/JS-2-2r	++	++	nd
						leukocytes	nd	-	nd
					Pig	PBEC	nd	+	nd

++ strong binding, + medium binding, +/- weak or heterogeneous binding, - no binding, nd not determined

Table S2: Chemical shifts (ppm)[a] of **Y9** in reduced form.

**Residue**	**NH**	**N**	**ND**	**NZ**	**HA**	**HB**	**HG**	**HD**	**HE**	**HZ**	**CA**	**CB**	**CG**	**CD**	**CE**	**CZ**
f-Met	8.52	129.1			4.46	1.93	2.45		2.06		54.2	33.1	31.8		16.9	
Ile	8.27	123.1			4.12	1.74	1.39	0.80			60.9	38.9	27.2	12.7		
							0.80						17.3			
Phe	8.58	125.8			4.65	3.06		7.25*	7.32*	7.26*	57.5	39.7		131.9	131.5	129.9
						2.98										
Leu	8.48	125.8			4.3	1.55	1.55	0.88			55.1	42.2	26.8	25.1		
								0.81						23.3		
Gly	7.86	109.0			3.83						45.3					
Gly	8.28	108.7			3.93						45.1					
Lys(PEG$_{27}$)	8.15	121.2		128.1	4.29	1.80	1.35	1.48	3.14	8.09	56.4	33.5	25.1	30.6	41.9	
						1.70										
Lys(PEG$_{27}$)	8.45	124.5		128.1	4.23	1.81	1.34	1.47	3.14	8.09	55.8	33.0	25.1	30.5	41.9	
						1.70										
D-Cys					4.23	3.10					57.0	27.9				
D-Asp	9.04	122.1			4.67	2.86					53.5	38.7				

						2.77									
Gly	8.47	109.8			3.83						45.1				
Tyr(3-NO_2)	8.21	118.6			4.72	3.21		7.96*	7.12*	7.95*	56.9	38.8		128.4*	122.6*
						2.98									
Gly	8.48	110.9			4.19						44.6				
					4.02										
Hyp					4.52	2.05*[b]	2.37*	3.78*			62.2	39.9*	39.9*	57.3	
								3.60*							
Asn	8.82	118.4	113.4		4.71	2.87		7.71			53.5	38.5			
						2.78		7.02							
D-Cys	8.19	119.6			4.43	2.89					58.8	28.4			

a. Spectra were obtained at pH 3.0 and 278 K and were referenced to the water signal.
b. * indicates side-chain resonances that could not be unambiguously assigned.

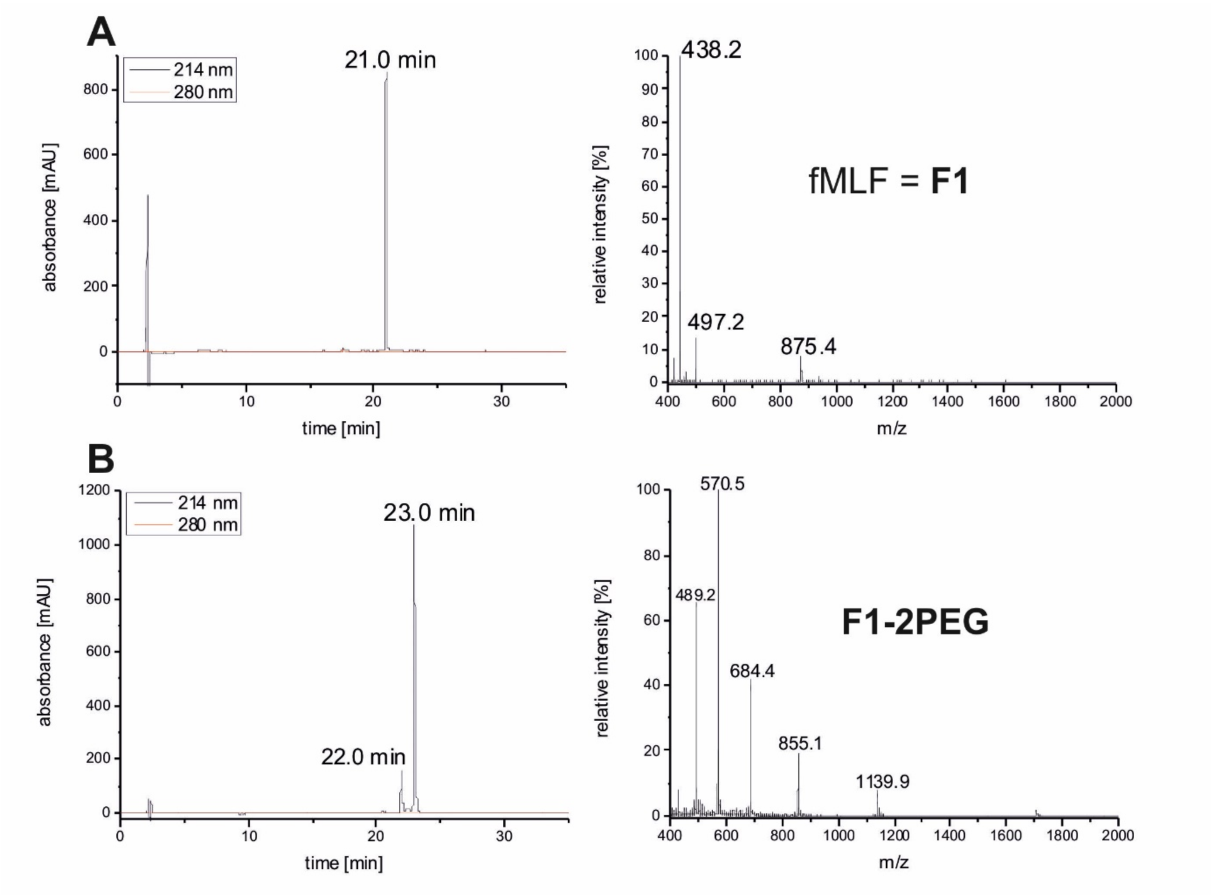

Figure S1: Synthesis data for F1 (A) and **F1-2PEG** (B). RP-HPLC chromatograms and ESI-MS spectra of purified material are shown. Calculated and observed molar masses of **F1** and **F1-2PEG** are in good agreement (**F1**: $MW_{calc.}$ = 437.6 Da, $MW_{obs.}$ = 438.2 Da; **F1-2PEG**: MW_{calc}. = 3417.1 Da, $MW_{obs.}$ = 3417.7 Da).

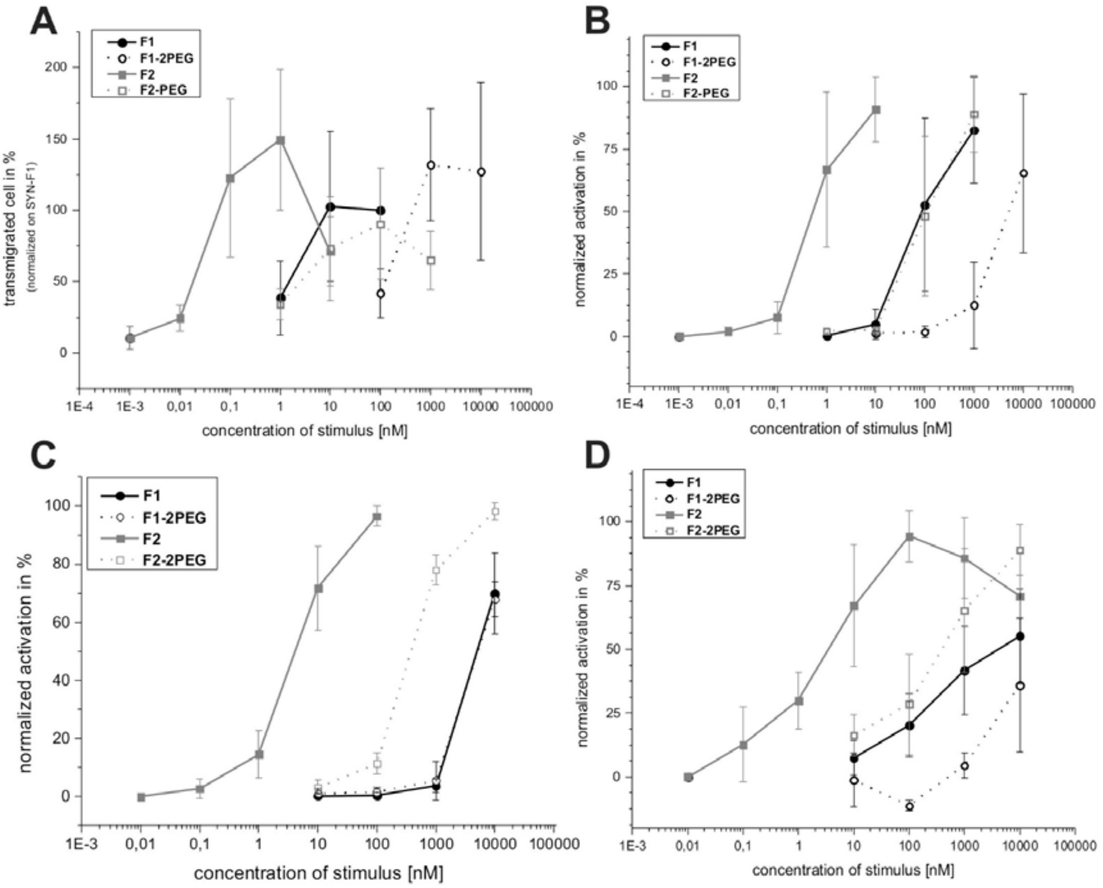

Figure S2: Comparison of effectors **F1** and **F2**. **A**) Chemotaxis of human leukocytes upon stimulation with different concentrations of (PEGylated) effectors. **B**) Oxidative burst of human leukocytes upon stimulation with (PEGylated) effectors. **C**) Oxidative burst of murine leukocytes upon stimulation with (PEGylated) effectors. **D**) Oxidative burst of guinea pig leukocytes upon stimulation with (PEGylated) effectors. In panels A-D, data of three independent experiments are averaged. The decrease of chemotaxis at high concentrations is a well-known observation as the receptors get saturated and no further movement along a gradient of effector is possible[2].

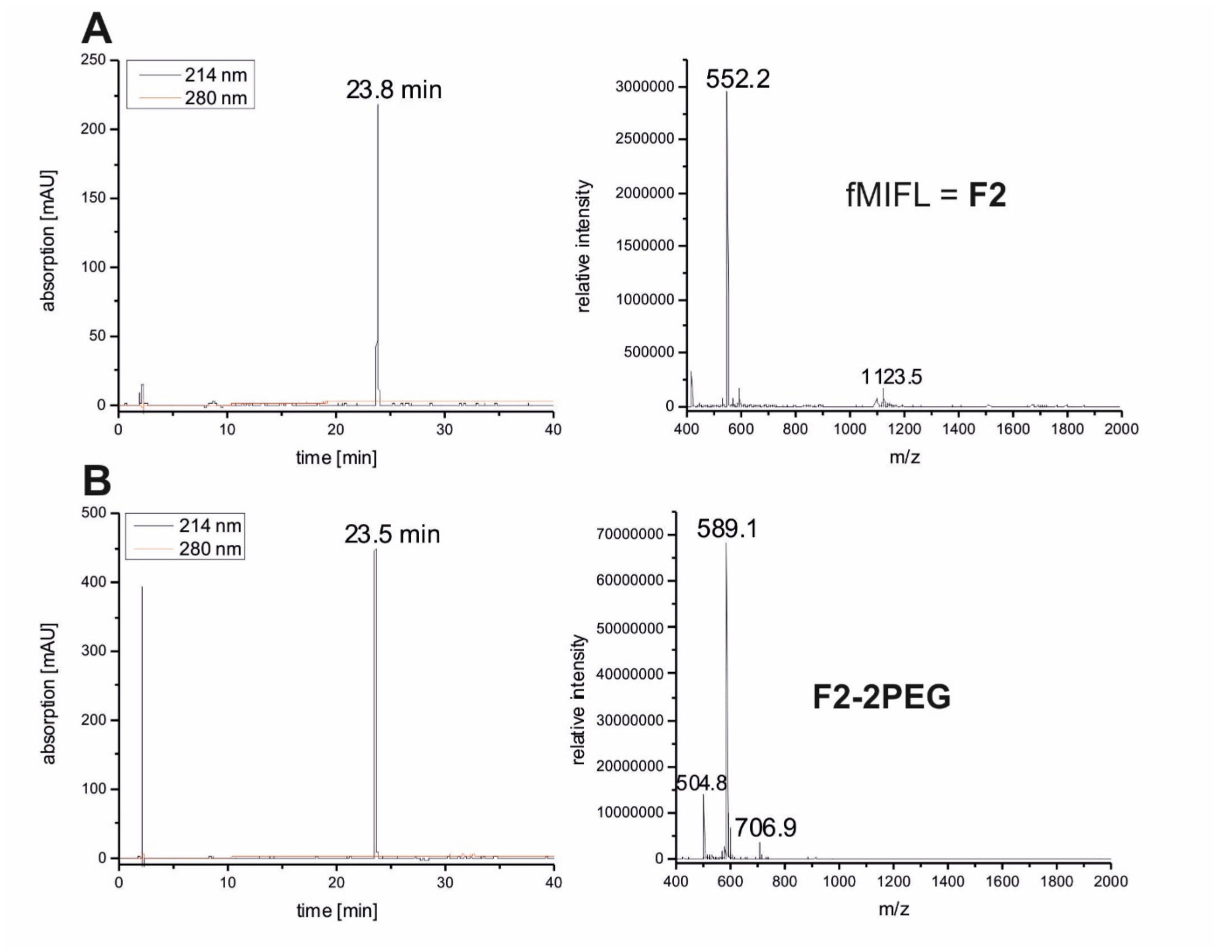

Figure S3: Synthesis data for **F2** (A) and **F2-2PEG** (B). RP-HPLC chromatograms and ESI-MS spectra of purified material are shown. Calculated and observed molar masses **F2** and **F2-2PEG** are in good agreement (**F2**: $MW_{calc.}$ = 550.7 Da, $MW_{obs.}$ = 551.3 Da; **F2-2PEG**: $MW_{calc.}$ = 3530.3 Da, $MW_{obs.}$ = 3529.0 Da).

.

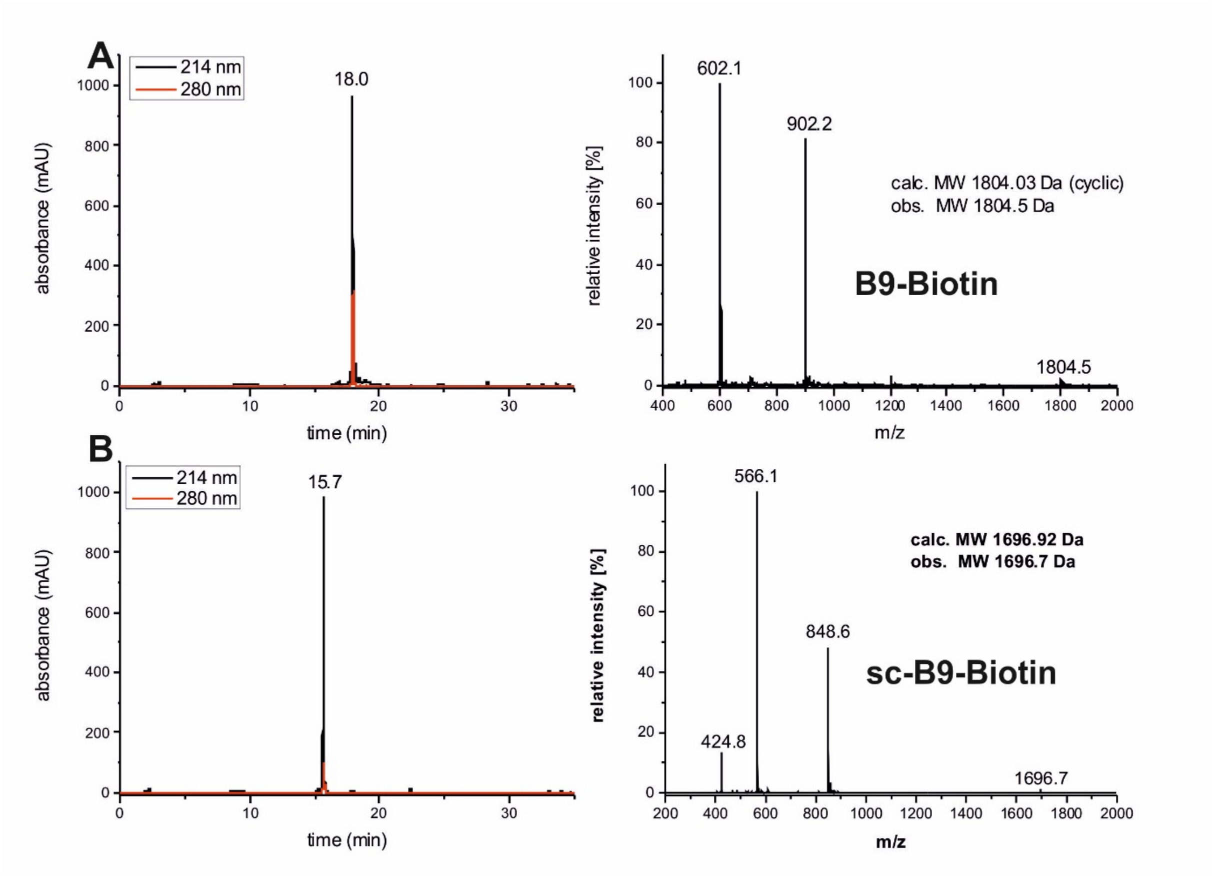

Figure S4: Synthesis data for **B9-Biotin** (A) and scrambled **B9-Biotin** (B). RP-HPLC chromatograms and ESI-MS spectra of purified material are shown. Calculated and observed molar masses as given in the figure are in good agreement with calculated values (for disulfide cyclized **Y9-Biotin**, **sc-B9** cannot cyclize).

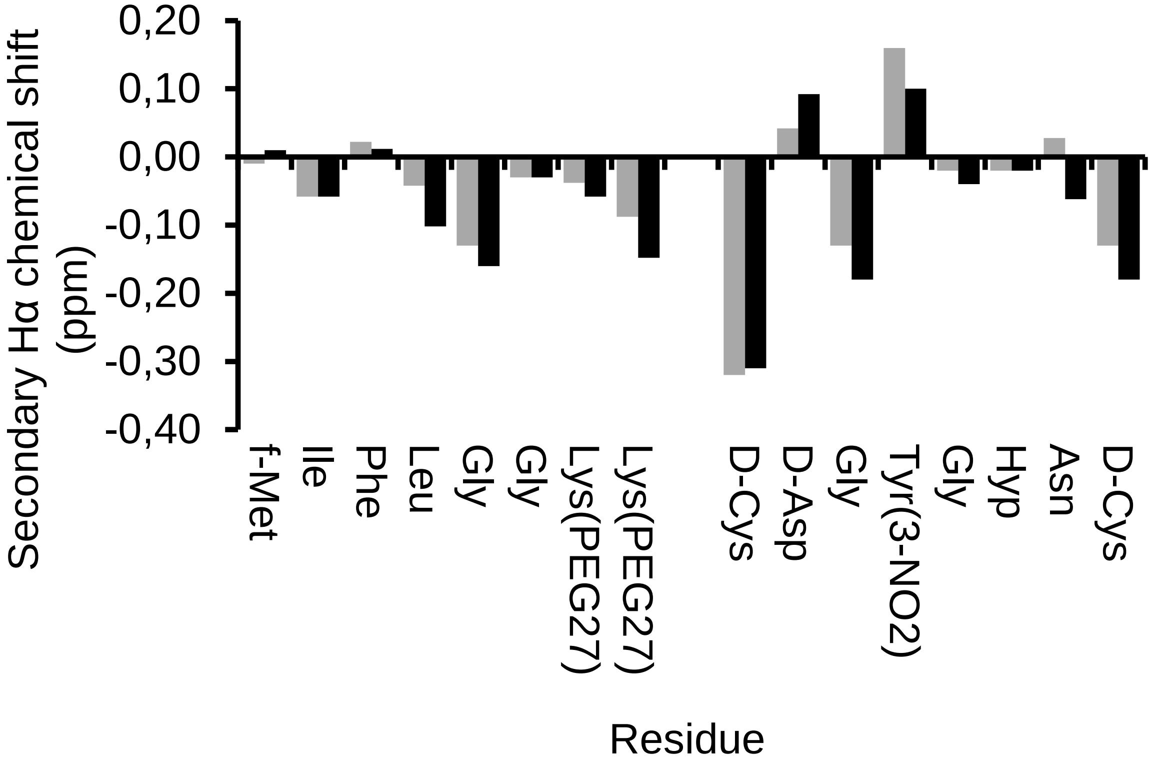

Figure S5: Secondary structural elements are typically indicated by the secondary Hα shifts; the difference between the Hα chemical shifts and their respective random coil shifts. Here the secondary Hα shifts of both the binder and effector peptides in **Y9** are < 0.1 ppm, indicating that both peptides are in a predominantly random coil conformation. Shifts for reduced **Y9** are shown in grey and for oxidized **Y9** in black.

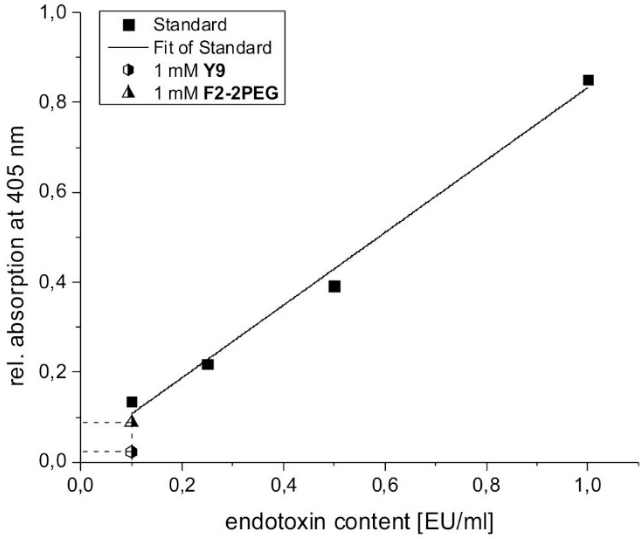

Figure S6: Endotoxin levels were determined in every batch of effector and **Y9** produced for this study to avoid any immune stimulatory effects of endotoxin impurities. A commercially available test system based on limulus amebocyte lysate was used here. Endotoxin levels in all products used were below the detection limit of 0.1 EU/ml.

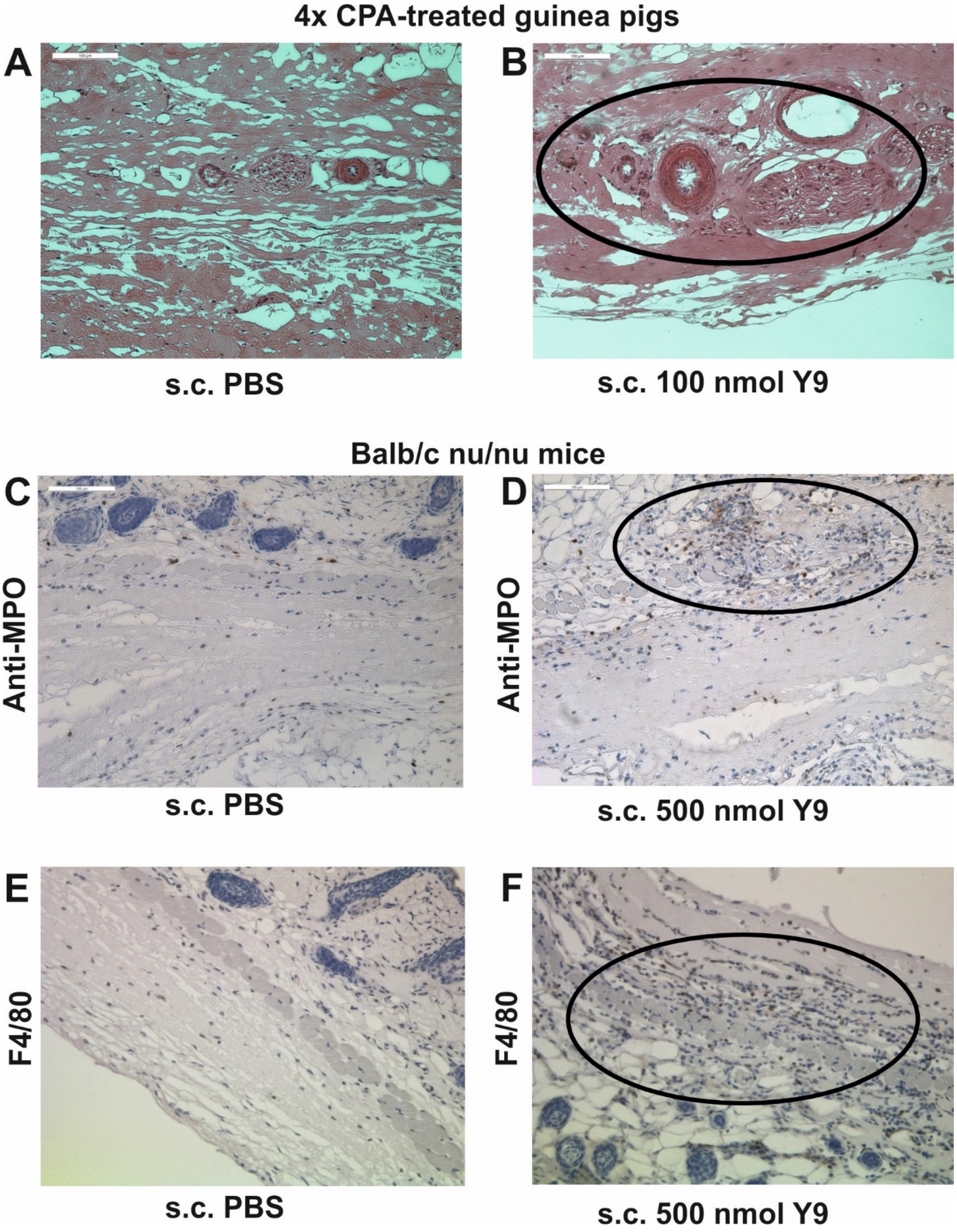

Figure S7: Hematoxylin and eosin (HE) stained skin sections (taken from injection sites) of guinea pigs (A, B) and immunohistochemically stained skin sections of Balb/c$^{nu/nu}$ mice (C-F) 24 h after subcutaneous injections of **Y9** and PBS (as control). **A, B**) Guinea pigs have been treated with CPA to suppress the immune system as required for GPC-16 tumor growth. **C, D**) Anti-myeloperoxidase (MPO) staining of murine skin sections. Brownish color indicates high concentrations of myeloperoxidase expressed in neutrophil granulocytes. **F, E**) Anti-F4/80 antibody staining of murine macrophages.

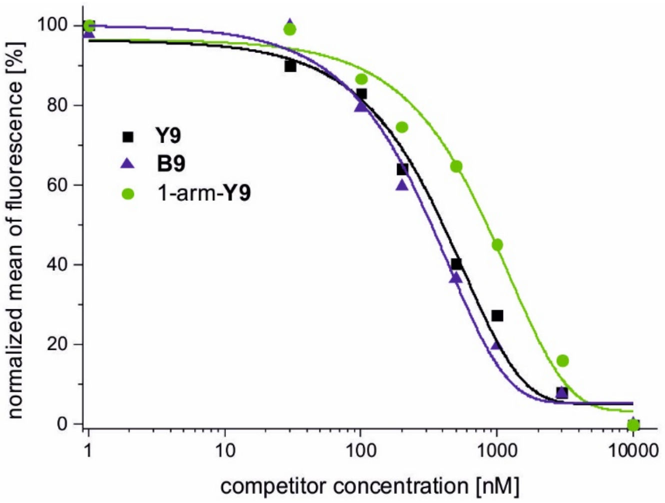

Binder	average IC_{50} ± SD [nM]
**Y9**	380 ± 120
1-arm-**Y9**	1533 ± 555
**B9**	318 ± 35

Figure S8: Competition of **B9**, **Y9** and 1-arm-**Y9** (Y9 carrying only one binder peptide) with 200 nM concentration of **B9-Biotin** on HT-29 cells in the presence of 2 mM $MnCl_2$. Data was fitted according to competition for one specific binding site and resulted in IC_{50} values using GraphPad Prism software (y = (Bmax*x)/(IC_{50}+x) + NS*x + background; NS = slope of nonlinear regression).

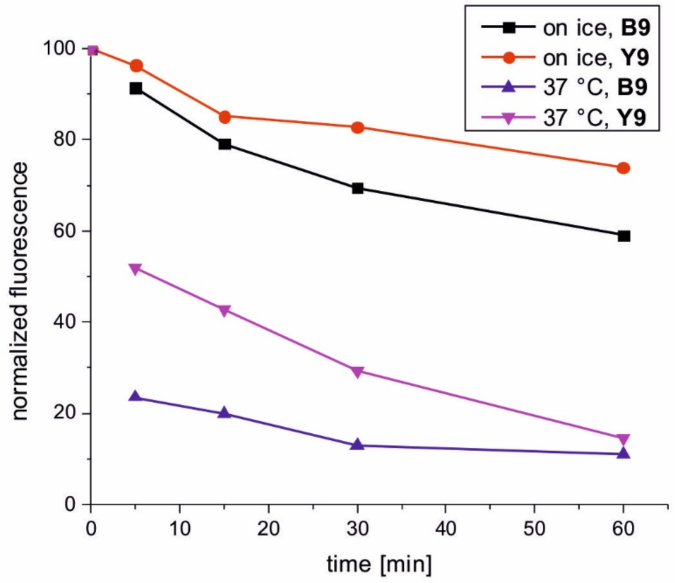

Figure S9: Flow cytometry analysis of the availability of biotinylated **B9** and **Y9** on PC-3 cells over time after incubation on ice and at 37°C. Biotinylated **B9** and **Y9** were detected with dye labeled streptavidin as described previously for flow cytometry assays.

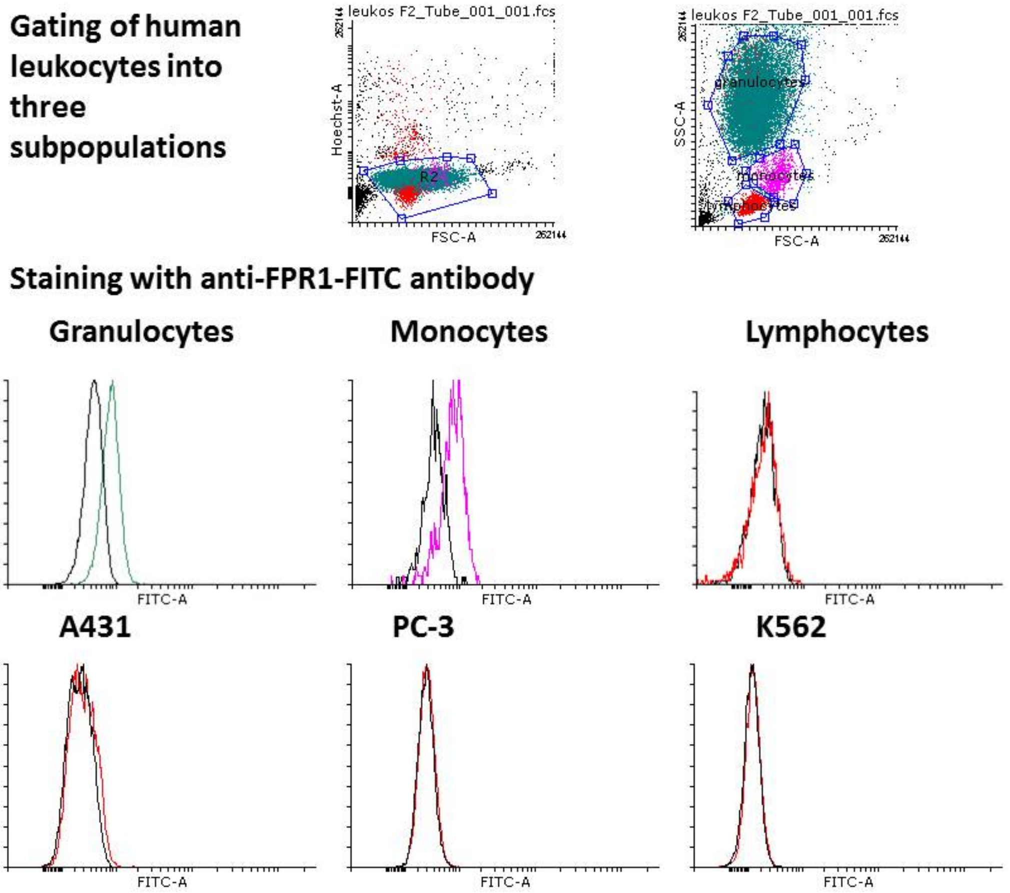

Figure S10: Binding of an FPR-1 specific antibody to human leukocytes. Top: Gating of human leukocytes for viability (R2 in the left hand panel) and into three subpopulations: granulocytes (green), monocytes (pink) and lymphocytes (red). Middle and bottom rows: Staining with anti-FPR-1 antibody (colored) versus isotype control (black). Human granulocytes and monocytes, but not lymphocytes express the FPR-1 receptor. None of the three tested human tumor cell lines express detectable levels of FPR-1.

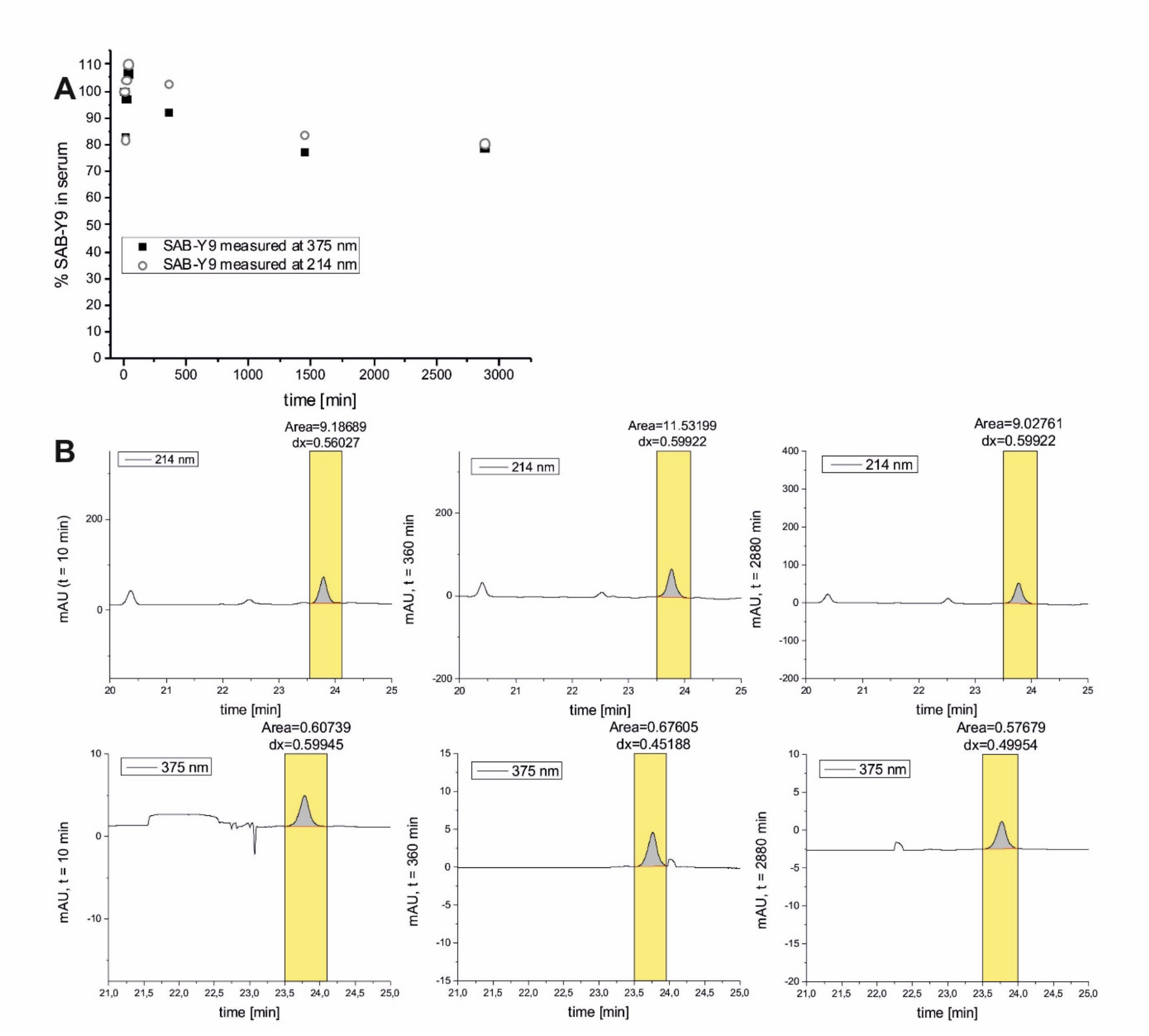

Figure S11: Serum stability of **Y9**. **A**) **Y9** was added to fresh mouse serum at 60 µM concentration and **Y9** was quantified after extraction from serum and subsequent RP-HPLC analysis (area under the peak). **B**) Selected chromatograms of **Y9** used for quantification by measurements at 214 and 375 nm and after 10, 360 and 2880 min.

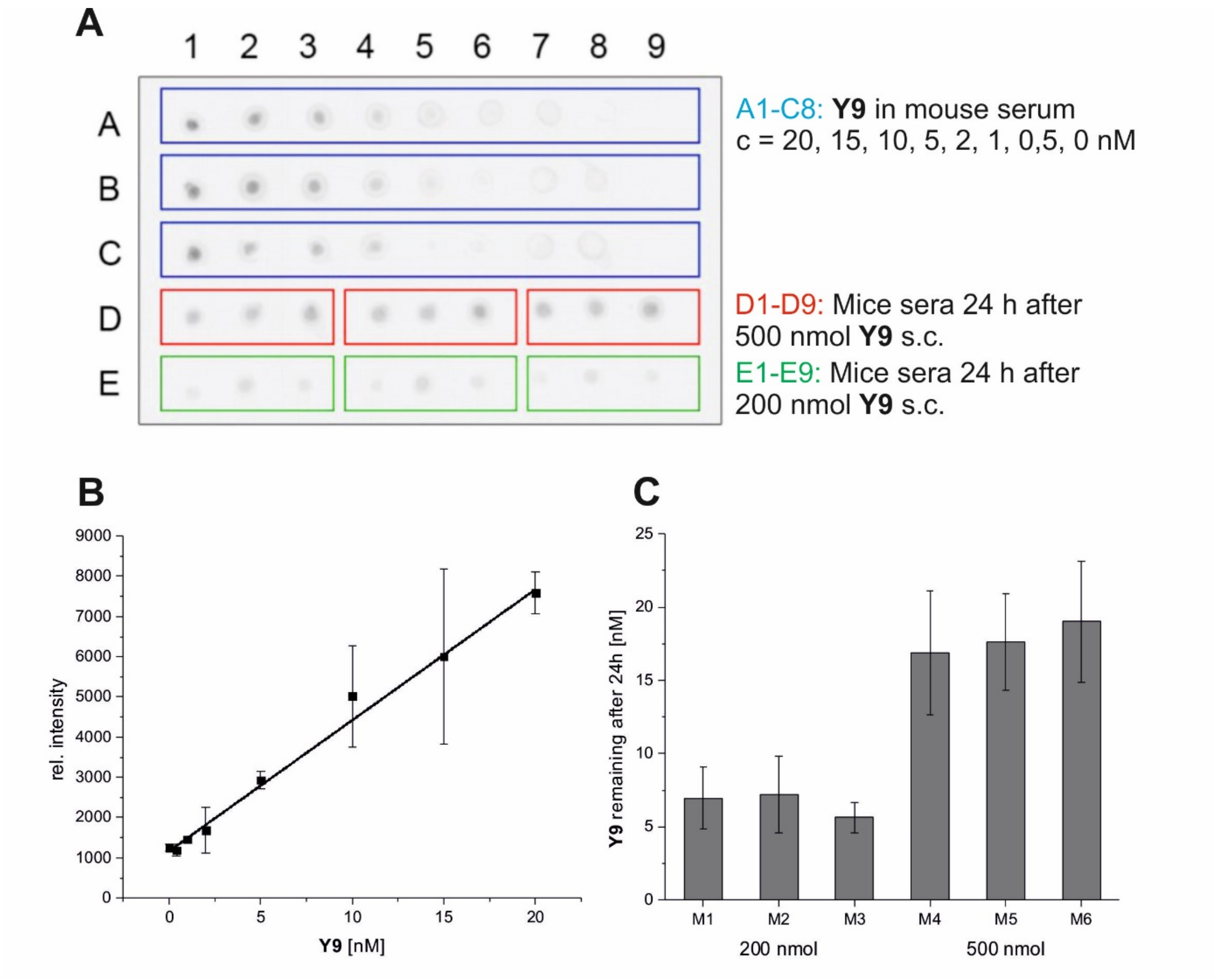

Figure S12: Dot-blot to determine **Y9** concentrations in mouse serum 24 h after s.c. injection of 200 or 500 nmol **Y9**, respectively. **A**) Original data. **B**) Calibration curve based on A1-C8. **C**) **Y9** concentration in 3 different mice for initial injection of 200 and 500 nmol.

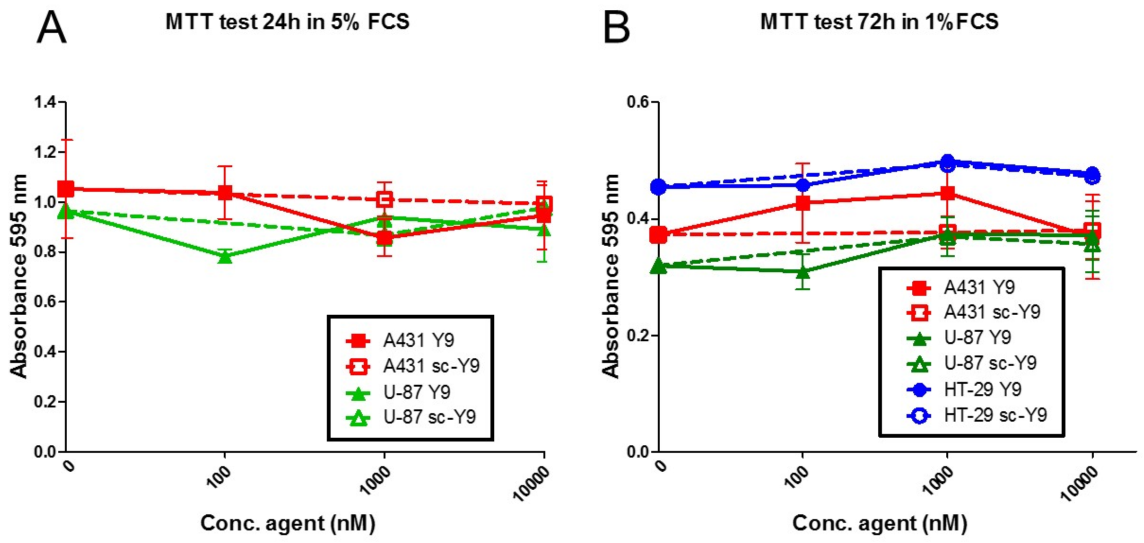

Figure S13: MTT assays showing no effect of **Y9** and **sc-Y9** on the viability and proliferation of different tumor cell lines. **A**) 24 h MTT assay with A431 and U-87 cells. **B**) 72 h MTT assay with A431, U-87 and HT-29 cells. Error bars represent the SDs of triple measurements. To indicate background: the absorbance values of medium without cells were between 0.11 and 0.13.

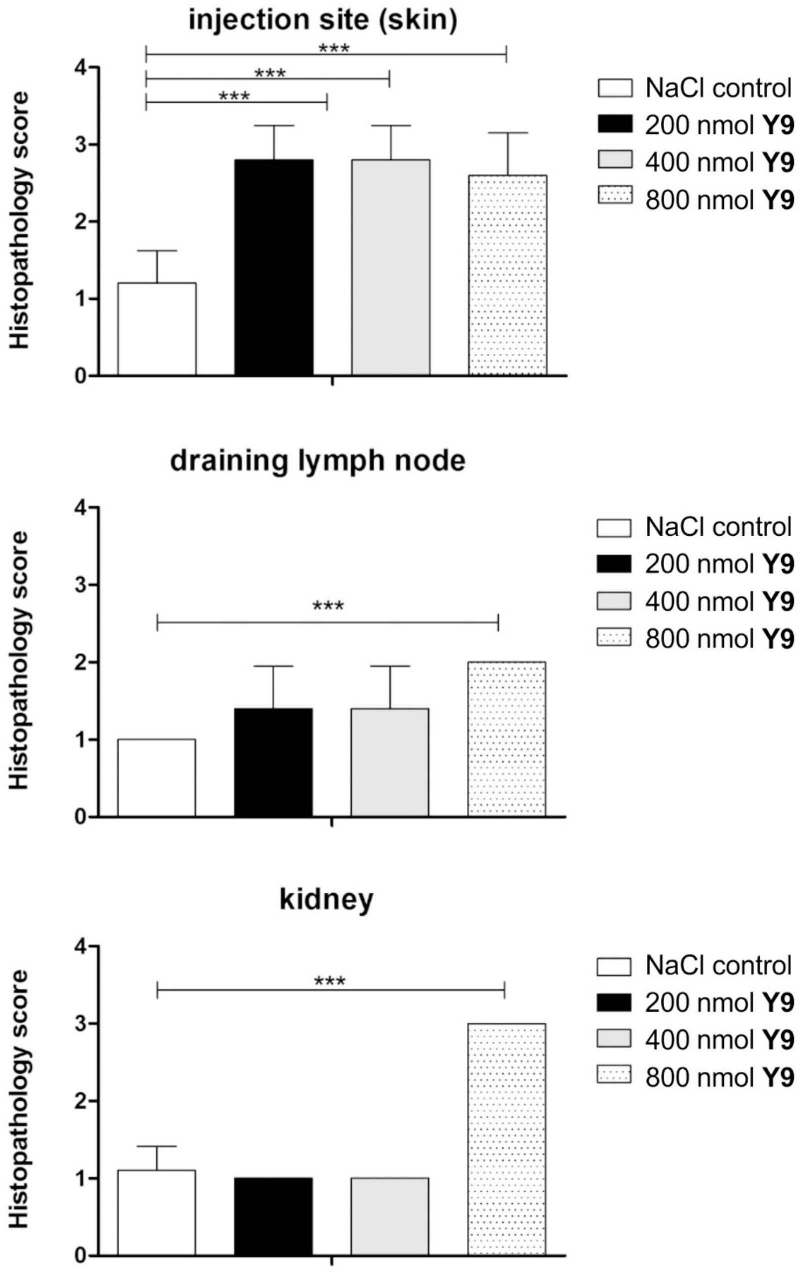

Figure S14: Histopathological evaluation of hematoxylin and eosin (HE) stained tissue sections of mouse organs 4 days after last (total of 7 injections every other day) subcutaneous injections of 200, 400 or 800 nmol of **Y9** and NaCl control. Organs (injection site (skin), liver, kidney, heart, brain, lung, pancreas, spleen, mesentery, draining and distant lymph nodes, ovaries, stomach, intestine) of 5 animals per **Y9** group and of 10 animals per NaCl control group were analyzed by a pathologist scoring the tissue damage/changes with 1=non or negligible, 2=mild, 3=moderate, and 4= severe comparison to the control group. Only statistically significant changes in histopathology of specific organs are shown. ***= $p<0.001$

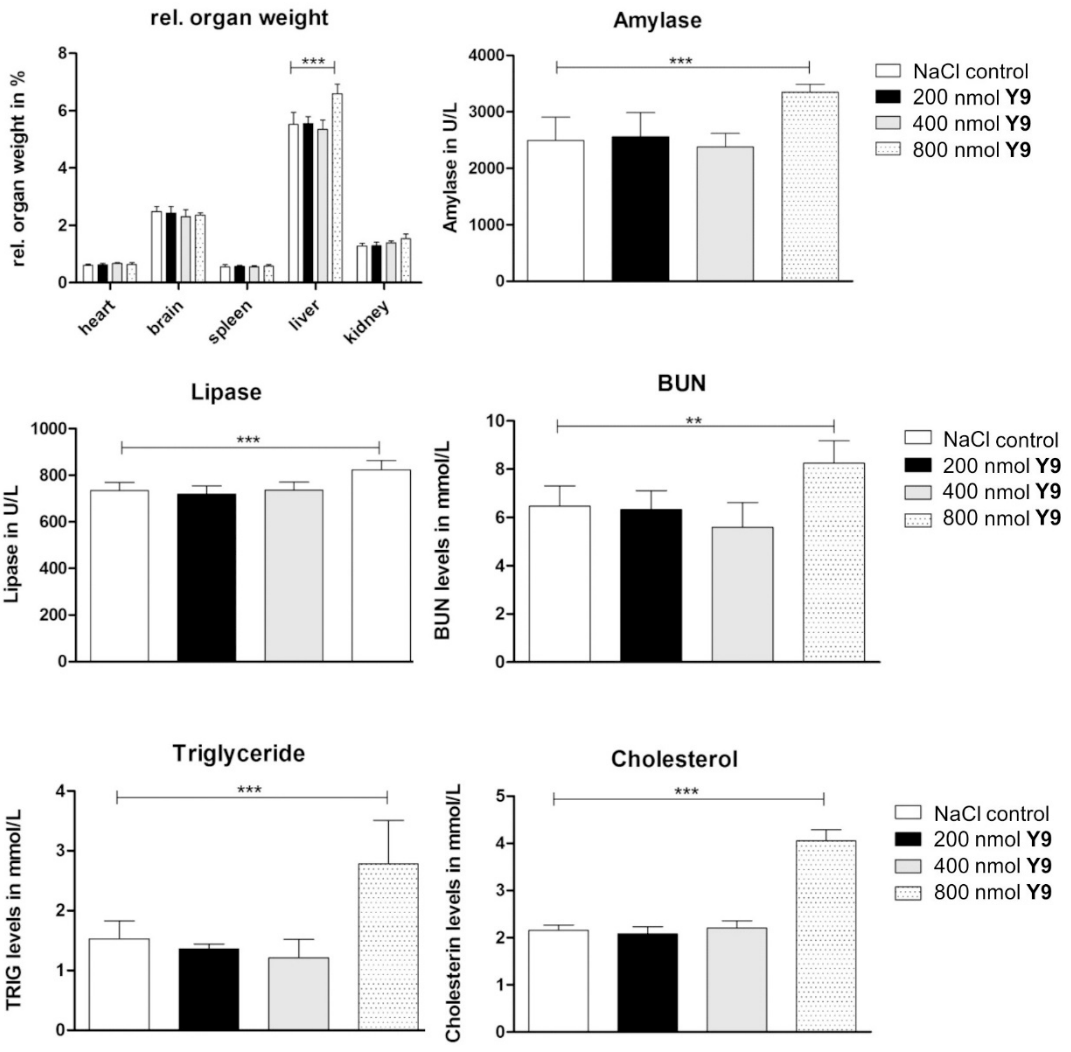

Figure S15: Relative organ weight measurement and serum analysis 4 days after last (total of 7 injections every other day) subcutaneous injections of 200, 400 or 800 nmol of **Y9** and NaCl control. Serum parameters (CK, ALP, Amylase, GOT/AST, GPT/ALT, GGT, LDH, Lipase, TP, GLU, CHOL, TRIG, BUN, CREA, TBIL, PHOS, Na, K, Ca, ALB) of 5 animals per **Y9** group and of 10 animals per NaCl control group were analyzed. Only statistically significant changes in serum parameters are shown. *** $p<0.001$; **$p<0.01$

References

1. Vowells, S.J., Sekhsaria, S., Malech, H.L., Shalit, M. & Fleisher, T.A. Flow cytometric analysis of the granulocyte respiratory burst: a comparison study of fluorescent probes. *J. Immunol. Methods* **178**, 89-97 (1995).

2. Niedel J., Wilkinson S. & Cuatrecasas P. Receptor-mediated uptake and degradation of 125-I-chemotactic peptide by human neutrophils. *J. Biol. Chem.* **254,** 10700-10706 (1979).
